# Decidual macrophage‐mediated ferroptosis in trophoblasts leads to recurrent spontaneous abortion

**DOI:** 10.1002/imt2.70138

**Published:** 2026-06-14

**Authors:** Xin Chen, Xueqin Ma, Pengcheng Pang, Heng Zhou, Ruohan Li, Yan He, Yan Zhang, Jing Yang, Qianlin Song, Qingsong Ye

**Affiliations:** ^1^ Department of Obstetrics and Gynecology Renmin Hospital of Wuhan University Wuhan Hubei China; ^2^ Central Laboratory Renmin Hospital of Wuhan University Wuhan Hubei China; ^3^ Center of Regenerative Medicine, Department of Stomatology Renmin Hospital of Wuhan University Wuhan Hubei China; ^4^ Faculty of Medicine and Health The University of Otago Dunedin New Zealand; ^5^ School of Stomatology Guangxi Medical University Nanning Guangxi China; ^6^ Reproductive Medical Center Renmin Hospital of Wuhan University and Hubei Clinic Research Center for Assisted Reproductive Technology and Embryonic Development Wuhan Hubei China; ^7^ Department of Urology Renmin Hospital of Wuhan University Wuhan Hubei China; ^8^ School of Dentistry The University of Sydney Sydney New South Wales Australia

**Keywords:** CXCL2/NF‐κB/HMOX1 axis, decidual macrophage, ferroptosis, recurrent spontaneous abortion, trophoblast cells

## Abstract

Recurrent spontaneous abortion (RSA) poses a significant challenge to successful early pregnancy, and trophoblast cell ferroptosis is an important pathogenic mechanism of RSA. However, it remains unclear whether decidual macrophages, as key immune regulators at the maternal–fetal interface, participate in the regulation of ferroptosis in trophoblast cells. This study observed significant ferroptosis in the placental trophoblast cells of patients with RSA and aborted mice. Transcriptomic sequencing results revealed that decidual macrophages derived from patients with RSA significantly promoted trophoblast cell ferroptosis while simultaneously impairing trophoblast cell function. Mechanistically, silencing heme oxygenase 1 (HMOX1) in trophoblast cells effectively reversed ferroptosis and restored trophoblast cell function, which was inhibited by decidual macrophages derived from patients with RSA. Notably, decidual macrophages regulate trophoblast ferroptosis and function by secreting C‐X‐C motif chemokine ligand 2 (CXCL2). Furthermore, the nuclear factor kappa‐B (NF‐κB) pathway was significantly enriched in trophoblast cells co‐cultured with decidual macrophages derived from patients with RSA. Further reversal experiments indicated that the CXCL2/NF‐κB/HMOX1 signaling axis may be a crucial mechanism by which decidual macrophages regulate trophoblast cell ferroptosis and function in RSA. Our subsequent findings demonstrated that trophoblast cells co‐cultured with RSA‐derived decidual macrophages promoted pro‐inflammatory polarization in macrophages. This effect was mediated by the interleukin‐6 (IL‐6) deficiency‐inhibited janus kinase 2 (JAK2)/signal transducer and activator of transcription 3 (STAT3) signaling axis. Finally, pharmacological analysis revealed Eriodictyol exhibits CXCL2‐axis‐associated protective effects in RSA. In conclusion, we observed that decidual macrophages in patients with RSA can induce ferroptosis in trophoblast cells, implying that targeting this mechanism may offer novel opportunities for reshaping maternal‐fetal tolerance.

## Highlights

- Decidual macrophages from recurrent spontaneous abortion (RSA) patients induce trophoblast ferroptosis and dysfunction through the CXCL2/NF‐κB/HMOX1 axis.
- Trophoblasts co‐cultured with RSA decidual macrophages promote pro‐inflammatory macrophage polarization through IL‐6 deficiency‐mediated inhibition of JAK2/STAT3 signaling.
- Eriodictyol shows therapeutic potential against RSA, offering a strategy to restore maternal‐fetal tolerance.

## Graphical abstract

Decidual macrophages from recurrent spontaneous abortion (RSA) patients promote trophoblast ferroptosis and impair trophoblast cell function. Mechanistically, the CXCL2/NF‐κB/HMOX1 axis mediates this effect. Conversely, trophoblasts co‐cultured with RSA decidual macrophages induce pro‐inflammatory macrophage polarization through IL‐6/JAK2/STAT3 signaling. Eriodictyol exhibits CXCL2‐axis‐associated protective effects in RSA. These findings identify decidual macrophage‐induced trophoblast ferroptosis as a key driver of RSA and suggest targeting this pathway may restore maternal‐fetal tolerance.

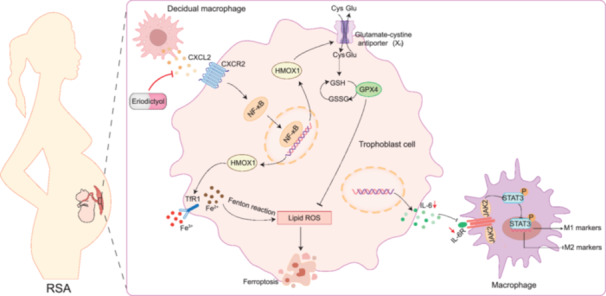

## INTRODUCTION

Recurrent spontaneous abortion (RSA) is a serious condition that poses a significant threat to the reproductive health of women and is characterized by the loss of two or more consecutive pregnancies with the same partner [1]. The incidence of RSA among women seeking conception is 1%–2% [2]. What is even more concerning is that the etiology of RSA is complex, with approximately 40%–50% of patients with RSA having unknown causes [1, 3]. The complex and unidentified etiology of RSA presents significant obstacles to its prevention and treatment. Therefore, research into the etiology of RSA is of great significance for its prevention, treatment, and drug development.

Ferroptosis is a distinct form of regulatory cell death that was first defined in 2012 [4]. The hallmarks of ferroptosis, an iron‐dependent programmed cell death, include excessive lipid peroxidation and reactive oxygen species (ROS) accumulation [5]. During placental development, tissue perfusion insufficiency and reperfusion, hypoxia‐reoxygenation [6], and ROS production often occur, significantly increasing the risk of trophoblast ferroptosis during pregnancy. Evidence suggests that trophoblast ferroptosis is closely associated with the onset of RSA. For instance, the inhibition of glutathione peroxidase 4 (GPX4) induces ferroptosis in human primary trophoblasts and placentas during pregnancy in mice [7]. Conversely, suppressing trophoblast ferroptosis can effectively improve pregnancy outcomes in mice with RSA [8]. Therefore, pharmacological inhibition of trophoblast ferroptosis may provide novel interventions for the treatment of RSA. However, the specific upstream mechanisms underlying trophoblast ferroptosis in RSA remain unclear.

Macrophages are pivotal immune components located at the maternal‐fetal interface, and maintaining an appropriate M1/M2 macrophage ratio is critical for a successful pregnancy. An imbalance in this ratio is associated with adverse pregnancy outcomes, including RSA, preeclampsia, and intrauterine growth restriction [9]. Our preliminary data revealed a predominance of M1‐type macrophages in the decidua of both patients with RSA and aborted mice. Inhibiting M1 macrophage polarization can effectively reduce the embryo absorption rate in mice [10]. Furthermore, macrophages may participate in the pathogenesis of RSA through crosstalk with trophoblast cells [11]. Our preliminary research suggests that macrophages can regulate the migration, invasion, proliferation, and apoptosis of trophoblast cells [10, 12]. Research has demonstrated that macrophages can alter the susceptibility of breast cancer cells to ferroptosis [13]. Another study found that macrophages can induce ferroptosis in cardiomyocytes via mitochondrial transfer [14]. However, the role of macrophages in regulating trophoblast ferroptosis during RSA remains to be elucidated.

This study aimed to explore the role of decidual macrophages in triggering trophoblast ferroptosis during RSA and elucidate the underlying mechanisms. Preliminary data suggest that in RSA, decidual macrophages secrete C‐X‐C motif chemokine ligand 2 (CXCL2), which interacts with C‐X‐C motif chemokine receptor 2 (CXCR2) on the surface of trophoblast cells, thereby activating the transcriptional activity of downstream nuclear factor kappa‐B (NF‐κB) to upregulate heme oxygenase 1 (HMOX1) expression, ultimately inducing trophoblast ferroptosis and contributing to RSA pathogenesis.

## RESULTS

### Ferroptosis is observed in the villous tissue of patients with RSA

**Figure 1:**
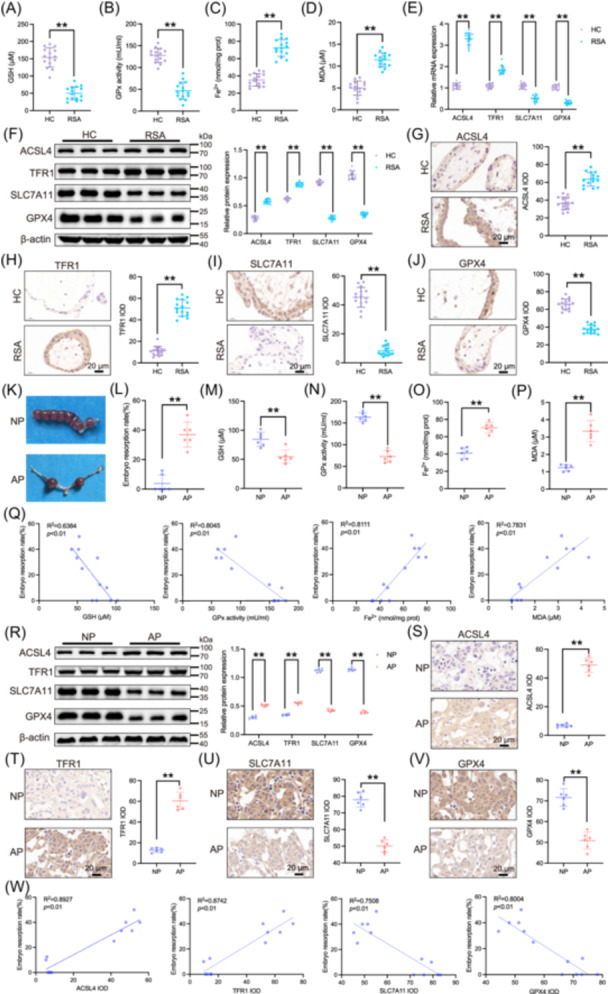
Ferroptosis is observed in the villous tissue of patients with recurrent spontaneous abortion (RSA). (A–D) Levels of glutathione (GSH), glutathione peroxidase (GPx) activity, Fe^2+^, and malondialdehyde (MDA) in villus tissue from patients with healthy control (HC) (*n* = 15) and RSA (*n* = 15). (E) To assess the expression of key ferroptosis markers, quantitative real‐time polymerase chain reaction (qRT‐PCR) was performed for acyl‐CoA synthetase long chain family member 4 (*ACSL4*), transferrin receptor 1(*TFR1*), solute carrier family 7 member 11 (*SLC7A11*), and glutathione peroxidase 4 (*GPX4*) mRNA from patients with HC (*n* = 15) and RSA (*n* = 15). (F) Western blot analysis and statistical values for ferroptosis markers ACSL4, TFR1, SLC7A11, and GPX4 from patients with HC (*n* = 15) and RSA (*n* = 15). (G–J) Expression of ferroptosis markers ACSL4, TFR1, SLC7A11, and GPX4 from patients with HC (*n* = 15) and RSA (*n* = 15) was detected by immunohistochemistry (IHC). (K, L) Embryo resorption rates in normal pregnancy (NP) and abortion pregnancy (AP) mice (*n* = 6 mice per group). (M–P) Levels of GSH, GPx activity, Fe^2+^, and MDA (*n* = 6 mice per group). (Q) Simple linear regression indicated that the embryonic resorption rate was significantly associated with placental GSH, GPx activity, Fe^2+^, and MDA (*n* = 12 mice). (R) Western blot analysis and statistical values for ferroptosis markers ACSL4, TFR1, SLC7A11, and GPX4 (*n* = 6 mice per group). (S–V) Expression of ferroptosis markers ACSL4, TFR1, SLC7A11, and GPX4 was detected by IHC (*n* = 6 mice per group). (W) Simple linear regression analysis revealed significant associations between embryo resorption rate and placental tissue levels of ferroptosis‐related proteins ACSL4, TFR1, SLC7A11, and GPX4 (*n* = 12 mice). Student's *t*‐test was employed for comparisons between two groups. **p < 0.01.

Ferroptosis is a distinct form of programmed cell death driven by iron and is characterized by the accumulation of ROS and lipid peroxidation. Glutathione (GSH), glutathione peroxidase (GPx) activity, Fe^2+^, and malondialdehyde (MDA) levels are widely recognized as key functional metabolic indicators of ferroptosis. To determine whether ferroptosis contributes to RSA progression, we first measured these indicators in villous tissue from patients with normal pregnancy (healthy control, HC group) and RSA (RSA group). The results revealed that patients with RSA exhibited significantly reduced GSH levels and GPx activity in villous tissue (Figure 1A,B), whereas Fe^2+^ and MDA levels were significantly increased (Figure 1C,D). Quantitative real‐time polymerase chain reaction (qRT‐PCR) and Western blot analyses were used to elucidate the molecular mechanisms underlying these observations. Compared to the HC group, patients with RSA exhibited upregulation of ferroptosis‐promoting proteins (acyl‐CoA synthetase long chain family member 4 (ACSL4) and transferrin receptor 1(TFR1)) and downregulation of ferroptosis‐inhibiting proteins (solute carrier family 7 member 11 (SLC7A11) and GPX4) in villous tissue (Figure 1E,F). The expression levels of these ferroptosis‐related proteins were subsequently examined using immunohistochemistry (IHC). These outcomes corroborated the data obtained from qRT‐PCR and Western blot analyses. Compared to the HC group, patients with RSA exhibited increased expression of the pro‐ferroptotic proteins ACSL4 and TFR1, along with reduced levels of the ferroptosis inhibitors SLC7A11 and GPX4 in villous tissues (Figure 1G–J). Furthermore, we developed mouse models representing normal pregnancy (NP) and abortion pregnancy (AP). The results revealed a significantly increased embryo resorption rate in the AP group (Figure 1K,L). We measured GSH, GPx activity, Fe^2+^, and MDA levels at the placental interface in the mice. The results revealed that AP mice exhibited significantly reduced GSH levels and GPx activity, with significantly elevated Fe^2+^ and MDA levels at the placental interface compared to NP mice (Figure 1M–P). Subsequently, simple linear regression analysis was used to examine the relationship between GSH, GPx activity, Fe^2+^, and MDA levels at the placental interface in mice and the rate of embryonic resorption. The levels of GSH and GPx activity in the placenta were negatively correlated with the embryonic resorption rate, whereas placental Fe^2+^ and MDA levels were positively correlated with the embryonic resorption rate (Figure 1Q). Western blot analysis of ferroptosis‐related protein expression revealed that AP mice demonstrated upregulation of pro‐ferroptosis proteins (ACSL4 and TFR1) and downregulation of anti‐ferroptosis proteins (SLC7A11 and GPX4) at the placental interface compared to NP mice (Figure 1R). IHC analysis further demonstrated the differential expression of key ferroptosis‐related proteins at the placental interface in the two groups. Compared to NP mice, AP mice exhibited elevated levels of the pro‐ferroptotic markers ACSL4 and TFR1, whereas the expression of the anti‐ferroptotic proteins SLC7A11 and GPX4 was decreased (Figure 1S–V). Finally, we analyzed the relationship between the levels of ferroptosis‐related proteins at the mouse placental interface and the embryo resorption rates. The results indicated that the expression of ferroptosis‐promoting proteins (ACSL4 and TFR1) in the mouse placenta was positively correlated with the embryo resorption rate, whereas the expression of ferroptosis‐inhibiting proteins, such as SLC7A11 and GPX4, in the mouse placenta was negatively correlated (Figure 1W). In summary, these findings underscore the role of ferroptosis in placental trophoblast cells in RSA pathophysiology.

### Decidual macrophages derived from patients with RSA cause ferroptosis in trophoblasts

**Figure 2:**
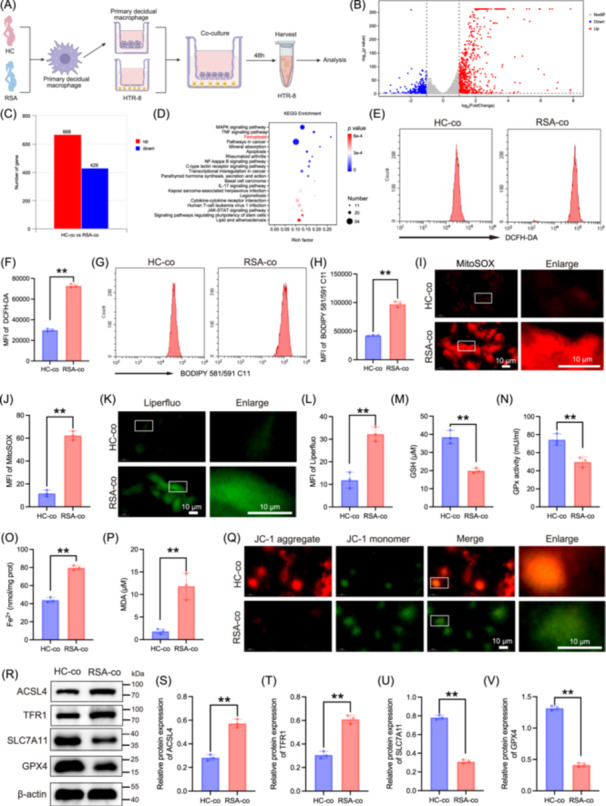
Decidual macrophages from recurrent spontaneous abortion (RSA) patients induce ferroptosis in trophoblasts. (A) Co‐culture of macrophages and trophoblasts was performed using a cell culture chamber. Human primary decidual macrophages from patients with healthy control (HC) (*n* = 3) and RSA (*n* = 3) were seeded in the upper chamber, while the HTR‐8/SVneo (HTR‐8) trophoblast cell line was seeded in the lower chamber and co‐cultured in a cell culture incubator for 48 h. (B) Transcriptome sequencing was performed on trophoblast cells co‐cultured with primary decidual macrophages from patients with HC (*n* = 3) and RSA (*n* = 3), and volcano plots showed upregulation and downregulation of genes. (C) The bar chart displays the number of upregulated and downregulated genes (*n* = 3). (D) Kyoto encyclopedia of genes and genomes (KEGG) pathway enrichment analysis (*n* = 3). (E, F) Cellular reactive oxygen species (ROS) levels were measured via flow cytometry based on the mean fluorescence intensity (MFI) of 2',7'‐Dichlorofluorescin diacetate (DCFH‐DA) (*n* = 3). (G, H) Lipid peroxidation was evaluated using BODIPY 581/591 C11 staining (*n* = 3). (I) Representative images of mitoSOX staining in trophoblast cells (*n* = 3). (J) Quantification of mitoSOX MFI in trophoblast cells (*n* = 3). (K, L) Intracellular lipid peroxidation was assessed using Liperfluo staining (*n* = 3). (M–P) Levels of glutathione (GSH), glutathione peroxidase (GPx) activity, Fe^2+^, and malondialdehyde (MDA) in trophoblast cells (*n* = 3). (Q) Assess mitochondrial membrane potential using JC‐1 staining (*n* = 3). (R–V) Western blot analysis and statistical values for ferroptosis markers acyl‐CoA synthetase long chain family member 4 (ACSL4), transferrin receptor 1(TFR1), solute carrier family 7 member 11 (SLC7A11), and glutathione peroxidase 4 (GPX4) (*n* = 3). Student's *t*‐test was employed for comparisons between two groups. **p < 0.01. HC‐co: trophoblast cells co‐cultured with primary decidual macrophages from patients with HC. RSA‐co: trophoblast cells co‐cultured with primary decidual macrophages from patients with RSA.

To investigate whether RSA‐derived decidual macrophages affect ferroptosis in trophoblast cells, we first collected placental tissues from patients in HC and RSA groups, isolated decidual tissues, and extracted primary decidual macrophages. Subsequently, we used a co‐culture system to implant primary decidual macrophages in the upper chamber and co‐cultured trophoblast cells in the lower chamber for 48 h. Transcriptome sequencing was performed on the co‐cultured trophoblast cells (Figure 2A). The volcano plot results indicated that decidual macrophages derived from patients with RSA significantly altered the transcription levels of trophoblast cells (Figure 2B,C). Kyoto encyclopedia of genes and genomes (KEGG) analysis revealed that the differentially expressed genes (DEGs) in trophoblast cells were significantly enriched in ferroptosis signaling pathways (Figure 2D). Subsequently, we measured ROS levels in both groups of trophoblast cells using the fluorescent probe 2',7'‐Dichlorofluorescin diacetate (DCFH‐DA) and assessed lipid peroxidation levels using BODIPY‐581/591 staining. Compared to controls, primary decidual macrophages obtained from patients with RSA significantly increased intracellular ROS levels in trophoblasts (Figure 2E,F) and promoted lipid peroxidation (Figure 2G,H). Simultaneously, we assessed mitochondrial superoxide (mitoSOX) production and lipid peroxidation levels using Liperfluo fluorescence. Compared to the controls, primary decidual macrophages from patients with RSA exhibited significantly increased mitoSOX production (Figure 2I,J) and lipid peroxidation levels (Figure 2K,L) within trophoblast cells. We also measured GSH, GPx activity, Fe^2+^, and MDA levels. Compared with controls, primary decidual macrophages from patients with RSA exhibited significantly reduced GSH levels and GPx activity within trophoblast cells, whereas Fe^2+^ and MDA levels were significantly increased (Figure 2M–P). Furthermore, JC‐1 staining revealed that primary decidual macrophages from patients with RSA exhibited a significantly reduced red (JC‐1aggregate)‐to‐green (JC‐1monomer) fluorescence ratio within trophoblast cells, indicating significant mitochondrial depolarization (Figure 2Q and Figure S1A). Finally, qRT‐PCR and Western blot analyses demonstrated that primary decidual macrophages obtained from patients with RSA upregulated the expression of pro‐ferroptotic markers (ACSL4 and TFR1) and downregulated anti‐ferroptotic markers (SLC7A11 and GPX4) in trophoblasts compared to controls (Figure S1B–E and Figure 2R–V). In summary, these results indicate that decidual macrophages from patients with RSA can significantly induce ferroptosis in trophoblast cells.

To explore the potential key role of decidual macrophages from patients with RSA in regulating trophoblast function, we assessed the proliferation capacity of the co‐cultured trophoblasts. Cell counting Kit‐8 (CCK‐8) and 5‐ethynyl‐2'‐deoxyuridine (EdU) experiments revealed that the proliferation capacity of trophoblasts co‐cultured with primary decidual macrophages from patients with RSA was significantly reduced compared to that of the control (Figure S2A,B). Similarly, terminal deoxynucleotidyl transferase‐mediated dUTP nick‐end labeling (TUNEL) staining results indicated that decidual macrophages from patients with RSA significantly enhanced trophoblast apoptosis (Figure S2C). Further flow cytometry analysis demonstrated that decidual macrophages from patients with RSA promoted trophoblast apoptosis compared to controls (Figure S2D). Wound‐healing assays demonstrated that the migration capacity of trophoblast cells significantly decreased following co‐culture with primary decidual macrophages derived from patients with RSA compared to the control group (Figure S2E). Furthermore, Transwell experiments indicated that decidual macrophages from patients with RSA significantly inhibited trophoblast cell invasion (Figure S2F,G). The qRT‐PCR and Western blot analyses confirmed alterations in key apoptotic and epithelial‐mesenchymal transition (EMT) markers. Compared to controls, co‐culture with primary decidual macrophages from patients with RSA significantly upregulated the mRNA and protein expression of the pro‐apoptotic marker BCL2‐associated X (BAX) in trophoblasts and downregulated the mRNA and protein expression of the anti‐apoptotic marker B‐cell lymphoma 2 (Bcl‐2) (Figure S2H,I). Decidual macrophages from patients with RSA concurrently upregulated E‐cadherin and downregulated Vimentin expression in trophoblasts (Figure S2H,I). In summary, these findings confirm that decidual macrophages from patients with RSA can suppress trophoblast function.

To investigate the role of ferroptosis in the inhibition of trophoblast function mediated by decidual macrophages from RSA patients, a series of rescue experiments was performed using the ferroptosis inhibitor Ferrostatin‐1 (Fer‐1). CCK‐8 and EdU assays demonstrated that the addition of Fer‐1 reversed the ability of RSA patient‐derived decidual macrophages to inhibit trophoblast proliferation (Figure S2J,K). TUNEL staining and flow cytometry revealed that the addition of Fer‐1 attenuated trophoblast apoptosis induced by RSA patient‐derived decidual macrophages (Figure S2L,M). Furthermore, Fer‐1 treatment significantly rescued the migration and invasion impairment in trophoblasts caused by decidual macrophages derived from patients with RSA (Figure S2N,O). Concurrently, qRT‐PCR and Western blot analyses confirmed that the altered expression of critical apoptotic (BAX and Bcl‐2) and EMT markers (E‐cadherin and Vimentin) was reversed by Fer‐1 treatment (Figure S2P,Q). These findings suggest that ferroptosis activation is a key mechanism underlying the adverse effects of RSA‐derived decidual macrophages on trophoblast function.

### RSA‐derived decidual macrophages regulate trophoblast ferroptosis via HMOX1

**Figure 3:**
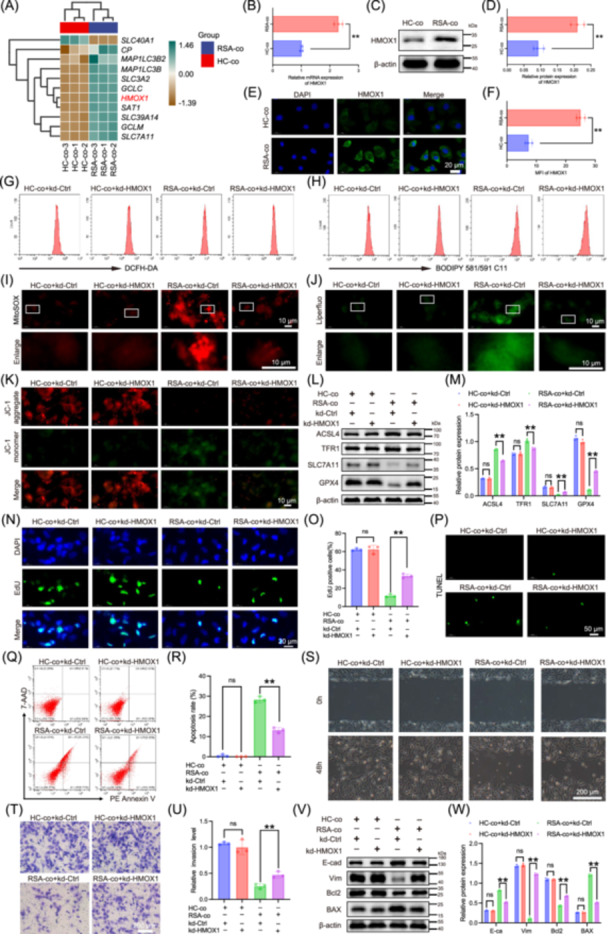
Recurrent spontaneous abortion (RSA)‐derived decidual macrophages regulate trophoblast ferroptosis via heme oxygenase 1 (HMOX1). (A) Heatmap illustrates genes differentially expressed in ferroptosis signaling pathways (*n* = 3). (B) Quantitative real‐time polymerase chain reaction (qRT‐PCR) results demonstrating *HMOX1* mRNA expression in both groups (*n* = 3). (C, D) HMOX1 protein expression in both groups was determined by Western blot analysis (*n* = 3). (E, F) Immunofluorescence results showing HMOX1 protein expression in both groups (*n* = 3). (G) Flow cytometry analysis of reactive oxygen species (ROS) levels (*n* = 3). (H) The BODIPY 581/591 C11 assay was utilized to evaluate lipid peroxidation levels (*n* = 3). (I) Representative images of mitoSOX staining in trophoblast cells (*n* = 3). (J) Intracellular lipid peroxidation assessed via Liperfluo staining (*n* = 3). (K) Mitochondrial membrane potential evaluated using JC‐1 staining (*n* = 3). (L, M) Western blot analysis and statistical values for key ferroptosis markers (acyl‐CoA synthetase long chain family member 4 (ACSL4), transferrin receptor 1(TFR1), solute carrier family 7 member 11 (SLC7A11), glutathione peroxidase 4 (GPX4)) (*n* = 3). (N, O) Cell proliferation assessed via 5‐ethynyl‐2'‐deoxyuridine (EdU) experiments (*n* = 3). (P) Apoptosis detected by terminal deoxynucleotidyl transferase‐mediated dUTP nick‐end labeling (TUNEL) staining (*n* = 3). (Q, R) Flow cytometric analysis and statistical results of cell apoptosis (*n* = 3). (S) Determination of trophoblast cell migration capacity via wound healing assay (*n* = 3). (T, U) Assessment of trophoblast cell invasion capacity via Transwell assay (*n* = 3). (V, W) Western blot analysis was performed to evaluate the expression of apoptosis‐related proteins (BCL2‐associated X (BAX) and B‐cell lymphoma 2 (Bcl‐2)) and epithelial‐mesenchymal transition (EMT) markers (E‐cadherin and Vimentin) (*n* = 3). Student's *t*‐test and one‐way analysis of variance were employed for comparisons between two groups and multiple groups, respectively. **p < 0.01, ns: not significant. E‐ca: E‐cadherin. Vim: Vimentin.

To further investigate the impact of decidual macrophages derived from patients with RSA on trophoblast ferroptosis, we focused on examining the DEGs enriched in the ferroptosis signaling pathway. Our analysis revealed that *HMOX1* exhibited the most significant differential expression among these genes (Figure 3A). To validate the role of *HMOX1*, we analyzed its expression in two groups of trophoblast cells. The results demonstrated a significant upregulation of *HMOX1* mRNA and protein levels in trophoblasts co‐cultured with RSA‐derived decidual macrophages compared to the control group (Figure 3B–D). Immunofluorescence results further confirmed that HMOX1 protein expression was significantly upregulated in trophoblast cells co‐cultured with decidual macrophages derived from patients with RSA (Figure 3E,F). To validate whether *HMOX1* is a crucial downstream gene in promoting trophoblast ferroptosis by decidual macrophages derived from patients with RSA, we knocked down *HMOX1* expression in trophoblasts (Figure S3A,B) and subsequently performed rescue experiments. The results indicated that *HMOX1* knockdown significantly reversed the pronounced increase in ROS production (Figure 3G and Figure S3C) and lipid peroxidation (Figure 3H and Figure S3D) in trophoblast cells derived from decidual macrophages isolated from patients with RSA compared to those in the control group. Simultaneously, we measured the production of mitoSOX and the degree of lipid peroxidation. The findings revealed that *HMOX1* knockdown reversed the promotion of mitoSOX production (Figure 3I and Figure S3E) and significantly increased lipid peroxidation (Figure 3J and Figure S3F) in trophoblast cells by primary decidual macrophages in patients with RSA. Furthermore, *HMOX1* knockdown reversed the reduction in GSH levels and GPx activity, as well as the elevation of Fe^2+^ and MDA levels induced by RSA‐derived decidual macrophages in trophoblasts (Figure S3G–J). JC‐1 staining revealed that *HMOX1* knockdown increased the red (JC‐1aggregate)‐to‐green (JC‐1monomer) fluorescence ratio within trophoblast cells, which was reduced by RSA‐derived decidual macrophages (Figure 3K and Figure S3L). Subsequently, qRT‐PCR and Western blot results revealed that *HMOX1* knockdown reversed the upregulation of ferroptosis‐promoting proteins ACSL4 and TFR1, as well as the downregulation of ferroptosis‐suppressing proteins SLC7A11 and GPX4, induced by RSA‐derived decidual macrophages in trophoblast cells (Figure S3K and Figure 3L,M). Based on the aforementioned findings, we observed that the effects of RSA‐derived decidual macrophages on trophoblast proliferation, apoptosis, migration, and invasion are closely associated with the activation of ferroptosis. We further examined alterations in trophoblast‐related functions. CCK8 and EdU assays revealed that *HMOX1* knockdown reversed the inhibitory effect of RSA‐derived decidual macrophages on trophoblast proliferation (Figure S3M and Figure 3N,O). TUNEL staining and flow cytometry revealed that *HMOX1* knockdown attenuated RSA‐derived decidual macrophage‐promoted trophoblast apoptosis (Figure 3P, Figure S3N, Figure 3Q,R). Furthermore, following *HMOX1* knockdown, the suppression of RSA‐derived decidual macrophage‐induced trophoblast migration (Figure 3S and Figure S3O) and invasion capacity (Figure 3T–U) was significantly attenuated. Concurrently, reversal of key apoptotic and EMT marker expression following *HMOX1* knockdown was confirmed by both qRT‐PCR and Western blot analyses (Figure S3P and Figure 3V–W). Collectively, these data indicate that decidual macrophages derived from RSA patients significantly upregulate HMOX1 expression in trophoblasts, thereby likely promoting trophoblast ferroptosis and suppressing trophoblast function.

### RSA‐derived decidual macrophages produce CXCL2 as a key regulator in the ferroptosis and other functions of trophoblast

**Figure 4:**
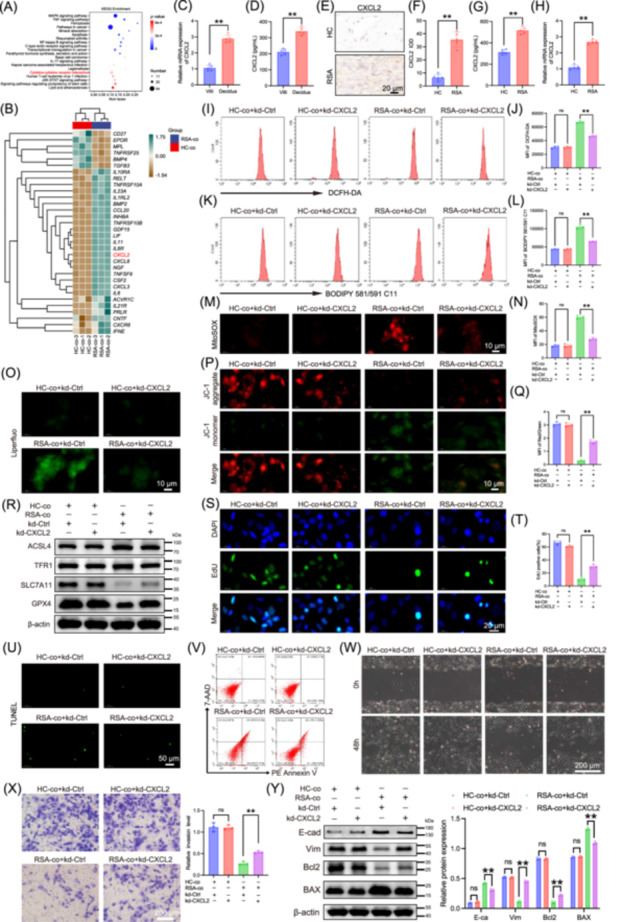
Recurrent spontaneous abortion (RSA)‐derived decidual macrophages produce C‐X‐C motif chemokine ligand 2 (CXCL2) as a key regulator in the ferroptosis and other functions of trophoblast. (A) Kyoto encyclopedia of genes and genomes (KEGG) bubble plot showing enriched signaling pathways of differentially expressed genes (*n* = 3). (B) Heatmap displaying differentially expressed genes enriched in the cytokine–cytokine receptor interaction pathway (*n* = 3). (C) Quantitative real‐time polymerase chain reaction (qRT‐PCR) detection of *CXCL2* expression in decidual and villous tissues from patients with healthy control (HC) (*n* = 5). (D) CXCL2 expression in decidual and villous tissues from patients with HC (*n* = 5) was quantified by ELISA. CXCL2 expression in decidual tissues from patients with HC (*n* = 5) and RSA (*n* = 5) was assessed by immunohistochemistry (IHC) (E, F), (G) enzyme‐linked immunosorbent assay (ELISA), (H) qRT‐PCR. (I, J) Flow cytometric analysis of reactive oxygen species (ROS) levels and statistical results (*n* = 3). (K, L) Lipid peroxidation was evaluated using BODIPY 581/591 C11 staining, with subsequent statistical analysis (*n* = 3). (M, N) Representative images of mitoSOX staining in trophoblast cells and statistical results (*n* = 3). (O) Intracellular lipid peroxidation assessed via Liperfluo staining (*n* = 3). (P, Q) Mitochondrial membrane potential was assessed by JC‐1 staining followed by statistical evaluation (*n* = 3). (R) Expression of ferroptosis‐related proteins (acyl‐CoA synthetase long chain family member 4 (ACSL4), transferrin receptor 1(TFR1), solute carrier family 7 member 11 (SLC7A11), glutathione peroxidase 4 (GPX4)) was analyzed by Western blot (*n* = 3). (S, T) The 5‐ethynyl‐2'‐deoxyuridine (EdU) assay was employed for the assessment of cell proliferation (*n* = 3). (U) Apoptosis was detected via terminal deoxynucleotidyl transferase‐mediated dUTP nick‐end labeling (TUNEL) staining (*n* = 3). (V) Flow cytometric analysis of cell apoptosis (*n* = 3). (W) Trophoblast migration was assessed by wound healing assay (*n* = 3). (X) Trophoblast cell invasion capacity was determined using Transwell assays (*n* = 3). (Y) Apoptosis‐related (BCL2‐associated X (BAX) and B‐cell lymphoma 2 (Bcl‐2)) and EMT‐related (E‐cadherin, Vimentin) protein expression was analyzed using Western blot (*n* = 3). Student's *t*‐test and one‐way analysis of variance were employed for comparisons between two groups and multiple groups, respectively. **p < 0.01, ns: not significant. E‐ca: E‐cadherin. Vim: Vimentin.

Next, to investigate the key mediators involved in trophoblast ferroptosis mediated by macrophage‐trophoblast crosstalk at the placental‐maternal interface in RSA, we performed further analysis of transcriptomic sequencing data. This revealed significant enrichment of the cytokine–cytokine receptor interaction signaling pathway (Figure 4A). Cytokines are important immune regulatory mediators secreted by macrophages. Subsequently, differentially expressed genes associated with the cytokine‐cytokine receptor interaction pathway were analyzed and we found that CXCL2 exhibited the most significant expression difference among them (Figure 4B). To further investigate the source of CXCL2, we examined its expression in decidual and villous tissues from HC patients. Results revealed significantly higher CXCL2 levels in decidual tissue compared to villous tissue (Figure 4C,D). This suggests that decidual macrophages originating from RSA may induce trophoblast ferroptosis by secreting CXCL2. We then validated CXCL2 expression using IHC, enzyme‐linked immunosorbent assay (ELISA), and qRT‐PCR. The RSA group demonstrated significantly higher CXCL2 levels in decidual tissues compared to the HC group (Figure 4E–H). These data indicate that CXCL2 is likely a key mediator through which decidual macrophages promote ferroptosis in trophoblasts. CXCL2 exerts its pathophysiological effects through the CXCR2 [15]. Therefore, CXCR2 expression was examined in trophoblast cells, revealing significant upregulation at both mRNA and protein levels following co‐culture with RSA patient‐derived decidual macrophages compared to controls (Figure S4A–C). To validate that CXCL2 secreted by decidual macrophages from RSA patients promotes key genes of ferroptosis in trophoblasts, we knocked down *CXCL2* expression in primary macrophages (Figure S4D,E) and performed rescue experiments. Compared to controls, *CXCL2* knockdown significantly reversed the marked increase in ROS (Figure 4I,J) and lipid peroxidation (Figure 4K,L) in trophoblast cells induced by primary decidual macrophages from RSA patients. Concurrently, we measured mitoSOX production and lipid peroxidation levels, revealing that *CXCL2* knockdown reversed the increase in mitoSOX production (Figure 4M,N) and lipid peroxidation (Figure 4O and Figure S4F) promoted by RSA‐derived decidual macrophages in trophoblast cells. Further results indicate that *CXCL2* knockdown reversed the reduction in GSH levels and GPx activity, as well as the increase in Fe^2+^ and MDA levels, promoted by RSA‐derived decidual macrophages within trophoblasts (Figure S4G–J). Furthermore, JC‐1 staining revealed that *CXCL2* knockdown elevated the red (JC‐1aggregate)‐to‐green (JC‐1monomer) fluorescence ratio in trophoblasts, which had been reduced by RSA‐derived decidual macrophages (Figure 4P,Q). Subsequent qRT‐PCR and Western blot analyses demonstrated that *CXCL2* knockdown reversed the upregulation of ferroptosis‐promoting proteins ACSL4 and TFR1, as well as the downregulation of ferroptosis‐suppressing proteins SLC7A11 and GPX4, induced by RSA‐derived decidual macrophages in trophoblast cells (Figure S4K, Figure 4R, and Figure S4L). We further examined alterations in trophoblast‐associated functions. CCK8 and EdU assays revealed that *CXCL2* knockdown reversed the inhibitory effect of RSA‐derived decidual macrophages on trophoblast proliferation (Figure S4M and Figure 4S,T). TUNEL staining and flow cytometry revealed that *CXCL2* knockdown mitigated RSA‐derived decidual macrophage‐promoted trophoblast apoptosis (Figure 4U, Figure S4N, Figure 4V, and Figure S4O). Furthermore, following *CXCL2* knockdown, the suppression of RSA‐derived decidual macrophage‐induced trophoblast migration (Figure 4W and Figure S4P) and invasion capacity (Figure 4X) was significantly attenuated. Concurrent analyses by qRT‐PCR and Western blot confirmed that *CXCL2* knockdown reversed the expression of pivotal apoptotic (BAX, Bcl‐2) and EMT (E‐cadherin, Vimentin) markers (Figure S4Q and Figure 4Y). These findings identify CXCL2 as a potential central regulator of trophoblast ferroptosis and dysfunction induced by RSA‐derived decidual macrophages.

### The NF‐κB signaling pathway mediates trophoblast ferroptosis induced by RSA‐derived decidual macrophages

**Figure 5:**
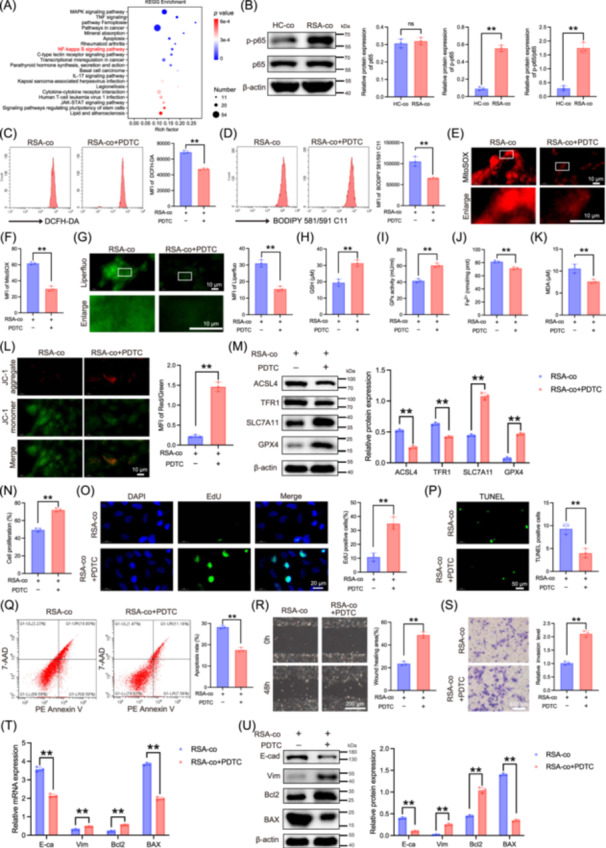
The nuclear factor kappa‐B (NF‐κB) signaling pathway mediates trophoblast ferroptosis induced by Recurrent spontaneous abortion (RSA)‐derived decidual macrophages. (A) Kyoto encyclopedia of genes and genomes (KEGG) bubble plot showing enriched signaling pathways among differentially expressed genes (*n* = 3). (B) NF‐κB pathway expression was compared in trophoblast cells by Western blot (*n* = 3). (C) Flow cytometry analysis of reactive oxygen species (ROS) levels and statistical results (*n* = 3). (D) Lipid peroxidation was quantified using BODIPY 581/591 C11 staining with subsequent statistical analysis (*n* = 3). (E, F) Analysis of mitoSOX staining and quantitative results in trophoblasts (*n* = 3). (G) Evaluation of intracellular lipid peroxidation via Liperfluo staining and statistical results (*n* = 3). (H–K) Levels of glutathione (GSH), glutathione peroxidase (GPx) activity, Fe^2+^, and malondialdehyde (MDA) in trophoblast cells (*n* = 3). (L) Mitochondrial membrane potential was evaluated by JC‐1 staining with statistical analysis (*n* = 3). (M) Expression levels of ferroptosis‐related proteins (acyl‐CoA synthetase long chain family member 4 (ACSL4), transferrin receptor 1(TFR1), solute carrier family 7 member 11 (SLC7A11), glutathione peroxidase 4 (GPX4)) were quantified by Western blot with statistical analysis (*n* = 3). (N) Cell proliferation assay using cell counting kit‐8 (CCK‐8) (*n* = 3). (O) Cell proliferation assay using 5‐ethynyl‐2'‐deoxyuridine (EdU) (*n* = 3). (P) Apoptosis detection via terminal deoxynucleotidyl transferase‐mediated dUTP nick‐end labeling (TUNEL) staining (*n* = 3). (Q) Flow cytometric analysis of cell apoptosis (*n* = 3). (R) Determination of trophoblast cell migration capacity via wound healing assay (*n* = 3). (S) Determination of trophoblast cell invasion capacity via Transwell assay (*n* = 3). (T) The mRNA expression of apoptosis markers BCL2‐associated X (*BAX*) and B‐cell lymphoma 2 (*Bcl‐2*), and epithelial‐mesenchymal transition (EMT) markers *E‐cadherin* and *Vimentin* was detected by quantitative real‐time polymerase chain reaction (qRT‐PCR) (*n* = 3). (U) The protein expression of apoptosis markers BAX and Bcl2, and EMT markers E‐cadherin and Vimentin was detected by Western blot (*n* = 3). Student's *t*‐test was employed for comparisons between two groups. **p < 0.01, ns: not significant. E‐ca: E‐cadherin. Vim: Vimentin.

Research indicates that CXCL2/CXCR2 signaling enhances the transcriptional activity of NF‐κB [16, 17]. Additionally, the promoter region of the *HMOX1* gene contains NF‐κB binding sites [18]. Sequencing analysis further demonstrated significant enrichment in the NF‐κB signaling pathway (Figure 5A). Therefore, to verify whether NF‐κB directly regulates *HMOX1* transcription, we performed Chromatin immunoprecipitation assay‐qPCR (CHIP‐qPCR) and dual luciferase reporter assays in trophoblast cells to detect the binding relationship between p65 (the core NF‐κB subunit) and the *HMOX1* promoter. CHIP‐qPCR experiments showed that p65 antibody enriched the *HMOX1* promoter sequence more than IgG, especially in trophoblast cells supplemented with CXCL2 (Figure S5A) or co‐culture with RSA‐derived decidual macrophages (Figure S5B). The luciferase reporter gene detection showed that overexpression of *p65* promoted the luciferase activity of reporter bodies carrying the *HMOX1* wild promoter, and this effect was eliminated by predicting mutations at the p65 binding site (Figure S5C). We therefore examined NF‐κB pathway expression in villous tissues from HC and RSA patients. Results showed markedly increased NF‐κB signaling in RSA villi compared to controls (Figure S6A,B). Subsequently, we examined NF‐κB signaling in trophoblast cells. Results revealed significantly elevated NF‐κB pathway expression in trophoblasts co‐cultured with decidual macrophages derived from RSA patients compared to controls (Figure 5B). We then performed a rescue experiment using the NF‐κB inhibitor pyrrolidinedithiocarbamate ammonium (PDTC ammonium) in trophoblast cells. PDTC ammonium treatment effectively counteracted the induced ROS overproduction (Figure 5C) and lipid peroxidation (Figure 5D) induced by RSA‐derived decidual macrophages in trophoblast cells compared to the control group. Similarly, PDTC ammonium intervention reversed the significant increase in mitoSOX production (Figure 5E,F) and lipid peroxidation levels (Figure 5G) promoted by RSA‐derived decidual macrophages in trophoblasts. Further results indicated that PDTC ammonium intervention reversed the significant reduction in GSH levels and GPx activity, along with the significant increase in Fe^2+^ and MDA levels, promoted by RSA‐derived decidual macrophages in trophoblasts (Figure 5H–K). JC‐1 staining further demonstrated that PDTC ammonium intervention elevated the ratio of red (JC‐1aggregate)‐to‐green (JC‐1monomer) fluorescence in trophoblast cells, which had been reduced by RSA‐derived decidual macrophages (Figure 5L). Additionally, qRT‐PCR and Western blot results demonstrated that PDTC ammonium intervention reversed the upregulation of ferroptosis‐promoting proteins ACSL4 and TFR1, as well as the downregulation of ferroptosis‐suppressing proteins SLC7A11 and GPX4, in trophoblasts induced by RSA‐derived decidual macrophages (Figure S7A–D and Figure 5M). Finally, we assessed alterations in trophoblast functions. CCK8 and EdU assays demonstrated that PDTC ammonium intervention reversed the suppression of proliferation capacity in trophoblasts induced by RSA‐derived decidual macrophages (Figure 5N–O). TUNEL staining and flow cytometry revealed that PDTC ammonium intervention mitigated RSA‐derived decidual macrophage‐induced trophoblast apoptosis (Figure 5P–Q). Furthermore, PDTC ammonium intervention significantly reduced the suppression of RSA‐derived decidual macrophage‐induced trophoblast migration (Figure 5R) and invasion capacity (Figure 5S). Concurrently, qRT‐PCR and Western blot analysis confirmed that PDTC ammonium intervention reversed the expression of key apoptotic and EMT markers, including BAX, Bcl2, E‐cadherin, and Vimentin (Figure 5T–U). These data indicate that the NF‐κB signaling pathway plays a crucial role in inducing ferroptosis in trophoblasts by RSA‐derived decidual macrophages.

To exclude off‐target effects of PDTC ammonium, we detected *HMOX1* mRNA and protein levels after knocking down *p65* (Figure S8A). The results showed that knocking down *p65* significantly reduced *HMOX1* mRNA and protein levels (Figure S8B,C). Subsequently, we further validated whether HMOX1 is a functional downstream effector of NF‐κB that drives ferroptosis and functional impairment in trophoblast cells. After overexpressing *HMOX1* (Figure S8D) in trophoblast cells that inhibit NF‐κB, we found that overexpression of *HMOX1* reversed the reduced ferroptosis and functional impairment of trophoblast cells by inhibiting NF‐κB (Figure S8E–P). In addition, we found that knocking down *CXCL2* in RSA derived decidual macrophages can reduce NF‐κB activation and HMOX1 expression in co‐cultured trophoblast cells (Figure S9A,B). Recombinant CXCL2 stimulation induces time‐dependent NF‐κB activation (Figure S9C) and subsequent upregulation of HMOX1 in trophoblast cells (Figure S9D), but this effect disappears due to *p65* knockdown (Figure S9E). Finally, our results showed that overexpression of *HMOX1* can reverse the inhibitory effect of *CXCL2* knockdown on ferroptosis in trophoblast cells and reverse the salvage effect of *CXCL2* knockdown on trophoblast cell function (Figure S9F–Q). It can be seen that HMOX1 is an indispensable downstream effector of CXCL2. In summary, our results suggest that the CXCL2/NF‐κB/HMOX1 signaling axis may be a key mechanism by which decidual macrophages regulate ferroptosis in trophoblast cells during RSA.

### RSA decidual macrophages train trophoblasts to modulate M1/M2 macrophages

**Figure 6:**
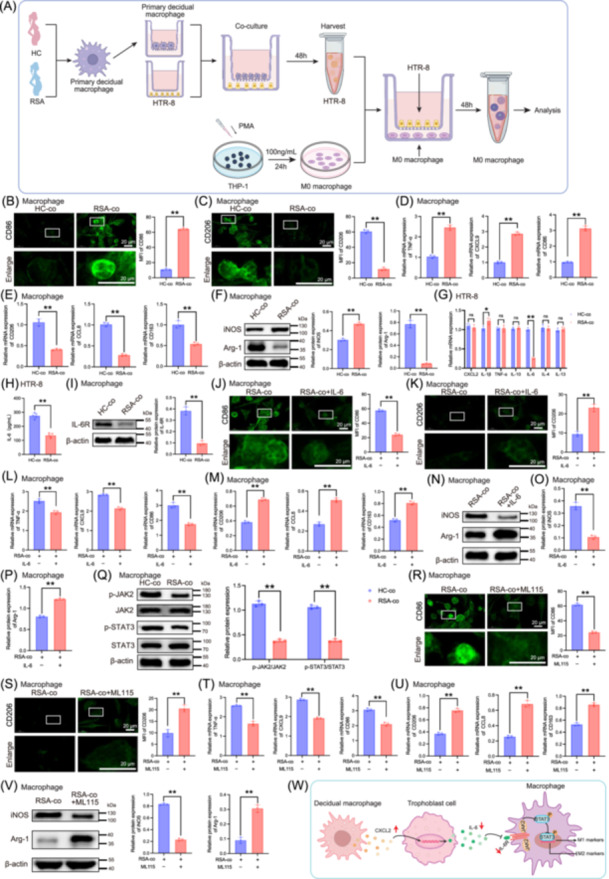
Recurrent spontaneous abortion (RSA) decidual macrophages train trophoblasts to modulate M1/M2 macrophages. (A) Co‐culture of trophoblast cells from patients with HC (*n* = 3) and RSA (*n* = 3) with decidual macrophages, followed by co‐culture with THP‐1‐derived macrophages, then detection of macrophages. (B) Representative fluorescence images of the M1 macrophage marker cluster of differentiation (CD)86 and quantified mean fluorescence intensity (MFI) of CD86 in macrophages (*n* = 3). (C) Representative fluorescence images of CD206, an M2 macrophage marker, and quantified MFI of CD206 in macrophages (*n* = 3). (D, E) The mRNA expression levels of M1 phenotype markers (tumor necrosis factor‐alpha (*TNF‐α*), CXC chemokine ligand 9 (*CXCL9*), and *CD86*) and M2 phenotype markers (*CD206*, C‐C Motif Chemokine Ligand (*8 CCL8*), and *CD163*) in macrophages were detected by qRT‐PCR (*n* = 3). (F) M1 (inducible nitric oxide synthase (iNOS)) and M2 (arginase‐1 (Arg‐1)) marker expression across experimental groups was analyzed by Western blot in macrophages (*n* = 3). (G) Differential expression of C‐X‐C motif chemokine ligand 2 (*CXCL2*), interleukin (*IL*)*−1β*, *TNF‐α*, *IL‐10*, *IL‐6*, *IL‐4*, and *IL‐13* in HTR‐8 cells was detected by quantitative real‐time polymerase chain reaction (qRT‐PCR) (*n* = 3). (H) IL‐6 expression was quantified by enzyme‐linked immunosorbent assay (ELISA) in HTR‐8 cells (*n* = 5). (I) Interleukin‐6 receptor (IL‐6R) protein expression in macrophages was quantified using Western blot (*n* = 3). (J) M1 macrophage marker CD86 expression visualized by immunofluorescence with corresponding MFI values in macrophages (*n* = 3). (K) M2 macrophage marker CD206 expression visualized by immunofluorescence with corresponding MFI values in macrophages (*n* = 3). (L, M) The qRT‐PCR analysis quantified M1 (*TNF‐α*, *CXCL9*, *CD86*) and M2 (*CD206*, *CCL8*, *CD163*) marker expression in macrophages (*n* = 3). (N–P) Western blot analysis detected M1 (iNOS) and M2 (Arg‐1) marker expression in macrophages (*n* = 3). (Q) Expression and phosphorylation levels of janus kinase 2 (JAK2)/signal transducer and activator of transcription 3 (STAT3) signaling were analyzed by Western blot with statistical quantification in macrophages (*n* = 3). (R) Immunofluorescence staining showing M1 marker CD86 expression with corresponding MFI in macrophages (*n* = 3). (S) Immunofluorescence staining showing M2 marker CD206 expression with corresponding MFI in macrophages (*n* = 3). (T, U) The qRT‐PCR analysis quantified M1 (*TNF‐α*, *CXCL9*, *CD86*) and M2 (*CD206*, *CCL8*, *CD163*) marker expression in macrophages (*n* = 3). (V) M1 (iNOS) and M2 (Arg‐1) marker expression in macrophages was analyzed by Western blot (*n* = 3). (W) Decidual macrophages from RSA patients educate trophoblastic cells to promote macrophage inflammatory activation by suppressing the JAK2/STAT3 axis via IL‐6. Student's *t*‐test was employed for comparisons between two groups. *p < 0.05, **p < 0.01, ns: not significant.

Our prior work revealed intricate interaction between trophoblasts and decidual macrophages throughout gestation [12, 19]. Concurrently, impaired macrophage function may lead to pathological pregnancies, such as RSA [10, 12]. To further investigate whether trophoblast cells co‐cultured with decidual macrophages from HC and RSA patients affect macrophage function. After co‐culturing with decidual macrophages derived from HC patients and RSA patients, trophoblasts were co‐cultured with tohoku hospital pediatrics‐1 (THP‐1) cells‐derived macrophages, followed by assessment of macrophage function (Figure 6A). We initially assessed macrophage polarization markers by immunofluorescence: cluster of differentiation (CD)86 (M1) and CD206 (M2). After co‐culturing with RSA‐derived decidual macrophages, trophoblast cells upregulated M1 marker CD86 (Figure 6B) and downregulated M2 marker CD206 (Figure 6C) in macrophages. This indicates that trophoblast cells co‐cultured with decidual macrophages from RSA patients promote M1 polarization while inhibiting M2 polarization in macrophages. To validate this finding, flow cytometry was employed to quantify M1 (CD86) and M2 (CD206) macrophage marker expression. We found that co cultured trophoblast cells with decidual macrophages from RSA patients significantly increased the expression of CD86 on macrophages (Figure S10A), but decreased the expression of CD206 (Figure S10B). The qRT‐PCR analysis of M1 (tumor necrosis factor‐alpha (*TNF‐α*), CXC chemokine ligand 9 (*CXCL9*), *CD86*) and M2 (*CD206*, C‐C Motif Chemokine Ligand 8 (*CCL8*), *CD163*) markers yielded results consistent with immunofluorescence and flow cytometry data (Figure 6D,E). Finally, protein immunoblotting for inducible nitric oxide synthase (iNOS) and arginase‐1 (Arg‐1) confirmed that the co‐culture of trophoblast cells with decidual macrophages from RSA patients significantly increased the expression of M1 macrophage marker iNOS, while inhibiting the expression of M2 marker Arg‐1 in macrophages (Figure 6F). In order to verify that the THP‐1 cell line derived macrophage cells can reproduce the phenotype and function of the primary human placental decidual macrophages that were the focus of this study, after co‐culturing with decidual macrophages derived from HC patients and RSA patients, trophoblasts were co‐cultured with primary human placental decidual macrophages, followed by assessment of macrophage M1 (TNF‐α, CXCL9, CD86) and M2 (CD206, CCL8, CD163) markers (Figure S11A). The results show that trophoblast cells co‐cultured with decidual macrophages from RSA patients promote decidual macrophage M1 polarization while inhibiting M2 polarization (Figure S11B). These findings suggest that trophoblast cells co‐cultured with decidual macrophages from RSA patients may influence macrophage polarization.

Trophoblast and macrophage cells can cross‐talk by producing and secreting cytokines or chemotactic molecules [20]. Therefore, we first detected the expression of molecules associated with macrophage polarization (C‐X‐C motif chemokine ligand 2 (*CXCL2*), Interleukin (*IL*)*−1β*, *TNF‐α*, *IL‐10*, *IL‐6*, *IL‐4*, and *IL‐13*) in trophoblast cells via qRT‐PCR. Expression levels of *IL‐1β* and *IL‐6* were altered, as determined by qRT‐PCR (Figure 6G), with the change in *IL‐6* being the most significant. Subsequently, we collected the supernatant from both groups of trophoblasts and quantitatively assessed IL‐6 expression via an ELISA. Results demonstrated a significant reduction in IL‐6 levels in the trophoblast supernatant following co‐culture with RSA‐derived decidual macrophages (Figure 6H). Concurrently, we examined expression of the IL‐6 receptor (IL‐6R) in macrophages. Results demonstrated that co‐cultured trophoblast cells with RSA‐derived decidual macrophages significantly inhibited the expression of interleukin‐6 receptor (IL‐6R) in macrophages (Figure 6I). These findings suggest that IL‐6 derived from trophoblast cells co‐cultured with decidual macrophages from RSA patients may influence macrophage polarization. Next, we added IL‐6 to or omitted IL‐6 from trophoblast cells co‐cultured with decidual macrophages derived from HC or RSA sources, then assessed macrophage polarization. Relative to the IL‐6‐free control, the IL‐6‐treated group exhibited suppressed M1 polarization and enhanced M2 polarization in macrophages (Figure 6J–P and Figure S10C,D). These findings indicate that trophoblast cells co‐cultured with RSA‐derived decidual macrophages can regulate macrophage polarization through IL‐6 secretion.

Emerging evidence underscores the pivotal role of the janus kinase 2 (JAK2)/signal transducer and activator of transcription 3 (STAT3) pathway in regulating macrophage inflammatory responses [21, 22]. To identify IL‐6‐responsive downstream signaling pathways in macrophages, we investigated the JAK2/STAT3 pathway. Revealed that cocultured trophoblast cells with decidual macrophages derived from RSA patients significantly suppressed the levels of phospho (p)‐JAK2/JAK2 and p‐STAT3/STAT3 proteins in macrophages (Figure 6Q). To further validate the role of JAK2/STAT3 signaling, we activated this pathway using ML115 (a STAT3 agonist) and subsequently assessed macrophage polarization. ML115 treatment shifted macrophage polarization from M1 to M2 phenotype compared to untreated controls (Figure 6R–V and Figure S10E,F). Collectively, these results confirm that co‐culturing with decidual macrophages from RSA patients enables trophoblast cells to regulate macrophage polarization by inhibiting the JAK2/STAT3 axis via IL‐6 deficiency (Figure 6W).

### The CXCL2/NF‐κB/HMOX1 signaling axis is critical for ferroptosis at the placental interface in aborted mouse

**Figure 7:**
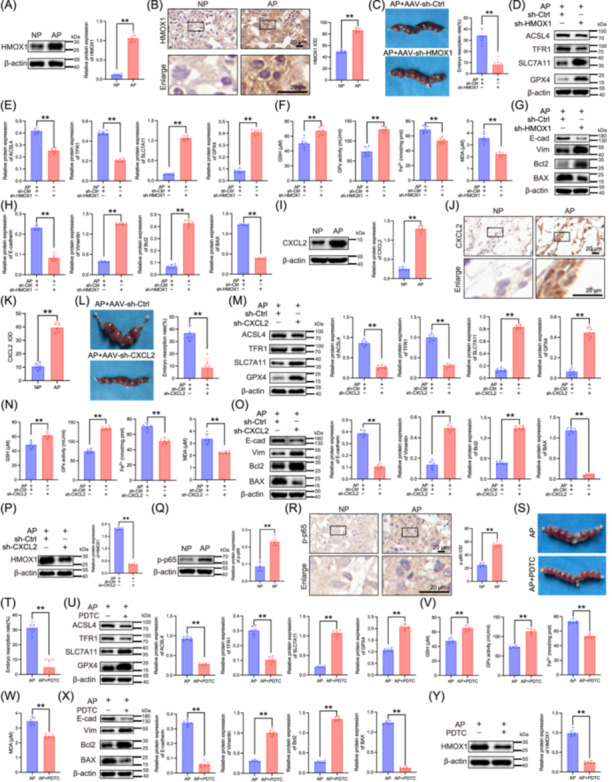
The C‐X‐C motif chemokine ligand 2 (CXCL2)/nuclear factor kappa‐B (NF‐κB)/heme oxygenase 1 (HMOX1) signaling axis is critical for ferroptosis at the placental interface in aborted mouse. (A) Verification of HMOX1 expression in placental tissues from normal pregnancy (NP) and aborted pregnancy (AP) mice via Western blot analysis (*n* = 6 mice per group). (B) Verification of HMOX1 expression in NP and AP placental tissues by immunohistochemistry (IHC) (*n* = 6 mice per group). (C) Embryo resorption rates in both groups following *HMOX1* knockdown intervention (*n* = 6 mice per group). (D, E) Expression and statistical analysis of ferroptosis‐related proteins: acyl‐CoA synthetase long chain family member 4 (ACSL4), transferrin receptor 1(TFR1), solute carrier family 7 member 11 (SLC7A11), and glutathione peroxidase 4 (GPX4), as determined by western blot (*n* = 6 mice per group). (F) Levels of glutathione (GSH), glutathione peroxidase (GPx) activity, Fe^2+^, and malondialdehyde (MDA) at the placental interface (*n* = 6 mice per group). (G,H) Western blot detection and statistical values of protein expression for apoptosis markers BCL2‐associated X (BAX) and B‐cell lymphoma 2 (Bcl‐2), and epithelial‐mesenchymal transition (EMT) markers E‐cadherin and Vimentin (*n* = 6 mice per group). (I) Validation of CXCL2 expression in NP and AP placental tissues by western blot (*n* = 6 mice per group). (J,K) Validation of CXCL2 expression in NP and AP placental tissues by IHC (*n* = 6 mice per group). (L) Embryo resorption rates in both groups following *CXCL2* knockdown intervention (*n* = 6 mice per group). (M) Expression and statistical analysis of ferroptosis‐related proteins: ACSL4, TFR1, SLC7A11, and GPX4, as determined by western blot (*n* = 6 mice per group). (N) Levels of GSH, GPx activity, Fe^2+^, and MDA at the placental interface (*n* = 6 mice per group). (O) Expression and statistical analysis of apoptosis (BAX, Bcl2) and EMT (E‐cadherin, Vimentin) markers as determined by Western blot (*n* = 6 mice per group). (P) Protein expression and statistical analysis of HMOX1 via Western blot (*n* = 6 mice per group). (Q) Validation of p‐p65 expression in NP and AP placental tissues by Western blot (*n* = 6 mice per group). (R) Immunohistochemical validation of p‐p65 expression in NP and AP placental tissues (*n* = 6 mice per group). (S, T) Embryo resorption rates in both groups after treatment with the NF‐κB signaling pathway inhibitor pyrrolidinedithiocarbamate (PDTC) ammonium (*n* = 6 mice per group). (U) Western blot analysis and statistical values for ferroptosis‐related proteins ACSL4, TFR1, SLC7A11, and GPX4 (*n* = 6 mice per group). (V, W) Levels of GSH, GPx activity, Fe^2+^, and MDA at the placental interface (*n* = 6 mice per group). (X) Protein expression and statistical analysis of apoptosis markers BAX and Bcl2, and EMT markers E‐cadherin and Vimentin via Western blot (*n* = 6 mice per group). (Y) Protein expression and statistical analysis of HMOX1 via Western blot (*n* = 6 mice per group). Student's *t*‐test was employed for comparisons between two groups. **p < 0.01. E‐ca: E‐cadherin. Vim: Vimentin.

To further elucidate the underlying mechanisms, we conducted in vivo studies using NP and AP mouse models. We first assessed HMOX1 expression at the placental interface in both groups via Western blot and IHC. Results revealed significantly higher HMOX1 expression in the placental interface of the AP group compared to the NP group (Figure 7A,B). Subsequently, we knocked down *HMOX1* in the aborted mice. Knockdown of *HMOX1* significantly reduced the elevated embryo resorption rate in AP group mice (Figure 7C). Western blot analysis indicated that *HMOX1* knockdown blocked the AP‐induced alterations in key ferroptosis regulators at the placental interface, notably reversing the upregulation of ACSL4 and TFR1 and the downregulation of SLC7A11 and GPX4 (Figure 7D,E). Further results indicated that *HMOX1* knockdown reversed the decreased GSH levels and GPx activity, along with the increased Fe^2+^ and MDA levels at the placental interface in AP group mice (Figure 7F). Concurrently, Western blot analysis showed that *HMOX1* knockdown normalized the altered expression levels of key apoptotic (BAX, Bcl2) and EMT (E‐cadherin, Vimentin) markers (Figure 7G,H). These findings collectively demonstrate that HMOX1 is a critical ferroptosis target in the placental interface. Similarly, we detected CXCL2 expression at the placental interface via Western blotting and IHC. AP group mice exhibited elevated CXCL2 expression at the placental interface relative to the NP group (Figure 7I–K). Subsequently, we knocked down *CXCL2* in the AP group. This intervention significantly reduced the embryo resorption rate (Figure 7L), suppressed ferroptosis (Figure 7M–N) and apoptosis (Figure 7O) at the placental interface in these mice. Furthermore, we observed that *CXCL2* knockdown reversed the elevated HMOX1 expression at the placental interface in the AP group mice (Figure 7P). Finally, we examined NF‐κB signaling pathway expression at the placental interface, revealing higher p‐p65 expression in the AP group mice compared to the NP group mice (Figure 7Q–R). Treatment with the NF‐κB pathway inhibitor PDTC ammonium significantly reversed embryo resorption rates in the AP group mice (Figure 7S–T), suppressed ferroptosis (Figure 7U–W) and apoptosis (Figure 7X) at the placental interface. Concurrently, PDTC ammonium treatment reversed the elevated HMOX1 expression at the placental interface in the AP group mice (Figure 7Y). Collectively, these findings indicate that the CXCL2/NF‐κB/HMOX1 signaling axis is critical for ferroptosis at the placental interface in aborted mice.

The findings from etiological studies on RSA must ultimately be translated into clinical practice. Traditional Chinese medicine (TCM) has an extensive history in managing RSA. Using the TCM‐information database (ID), we screened and identified 10 herbal components targeting aborted, through further cavity‐detection guided blind docking (CB‐Dock) 2 docking simulations, we identified Eriodictyol as the compound exhibiting the highest binding affinity for CXCL2 (Figure S13A,B). Eriodictyol has been demonstrated to be beneficial in the treatment of RSA [23]. Next, we attempted to treat the AP group mice with Eriodictyol, and the results showed that Eriodictyol treatment significantly reversed the embryo resorption rate in the AP group mice (Figure S13C,D). We subsequently examined CXCL2 expression at the placental interface and found that Eriodictyol significantly suppressed it in the AP group (Figure S13E–H). Western blot analysis further demonstrated that eriodictyol treatment in AP group mice counteracted the aberrant expression of ferroptosis‐related proteins at the placental interface by reducing elevated ACSL4 and TFR1 levels while restoring diminished SLC7A11 and GPX4 expression (Figure S13I–M). Further results indicate that Eriodictyol treatment reversed the reduction in GSH levels and GPx activity, as well as the elevation in Fe^2+^ and MDA levels, within the placental interface of the AP group mice (Figure S13N–Q). Western blot analysis further demonstrated Eriodictyol's regulatory effects on critical apoptotic and EMT markers, showing restored expression patterns of BAX, Bcl‐2, E‐cadherin, and Vimentin (Figure S13R–V). These findings collectively indicate that Eriodictyol's protective action against RSA may be mediated through CXCL2 regulation.

To further verify whether the mechanism of action of Eriodictyol in treating RSA is through the CXCL2/NF‐κB/HMOX1 axis, we first intervened with Eriodictyol in the co‐culture model and found that Eriodictyol blocked the binding of CXCL2‐CXCR2 (Figure S14A) and the activation of downstream signaling pathways (Figure S14B). Subsequently, we validated *in vitro* whether overexpression of *CXCL2* or *HMOX1* and activation of NF‐κB would eliminate the protective effect of Eriodictyol. The in vitro model found that overexpression of *CXCL2* or *HMOX1* and intervention of NF‐κB activator 1 reversed ferroptosis and apoptosis inhibited by Eriodictyol (Figure S14C–I). Similarly, in an in vivo mouse model, overexpression of *CXCL2* or *HMOX1* and intervention with NF‐κB activator 1 reversed the increase in embryonic resorption rate inhibited by Eriodictyol (Figure S14J–L). In summary, our results indicate that Eriodictyol exerts its inhibitory effect on miscarriage through the CXCL2/NF‐κB/HMOX1 axis, rather than through non‐specific antioxidant/anti‐inflammatory effects.

## DISCUSSION

The results of this study indicate that significant ferroptosis occurs in the trophoblast cells of placentas from patients with RSA and from aborted mouse fetuses. Further transcriptomic sequencing revealed that decidual macrophages derived from RSA patients significantly promoted ferroptosis in trophoblast cells, simultaneously impairing their proliferation, migration, and invasion capabilities while accelerating trophoblast apoptosis. Mechanistically, the CXCL2/NF‐κB/HMOX1 signaling axis may represent a key pathway through which decidual macrophages regulate trophoblast ferroptosis and function in RSA. Subsequently, we found that trophoblast cells co‐cultured with RSA‐derived decidual macrophages exhibited a pro‐inflammatory polarization effect on macrophages, mediated through IL‐6 inhibition of the JAK2/STAT3 pathway. Finally, pharmacological analysis demonstrated Eriodictyol exhibits CXCL2‐axis‐associated protective effects in RSA.

As normal trophoblast function is crucial for embryo implantation, placental formation, and establishing maternal‐fetal circulation, its impairment may result in RSA [24]. Trophoblast cells are rich in polyunsaturated fatty acids and are susceptible to oxidative stress, making them prone to ferroptosis, and trophoblast ferroptosis may contribute to the pathogenesis of placental disorders, including RSA [6, 25, 26]. Research utilizing an oxidative stress‐induced rat model of aborted pregnancy revealed that, compared to healthy non‐pregnant rats, the trophoblast cells of aborted rats exhibited decreased GPX4 levels alongside elevated TFR1 and ACSL4 levels, this demonstrates the presence of ferroptosis in the rat model of aborted pregnancy [26]. Compared to healthy controls, previous investigations have demonstrated that the expression of hypoxia inducible factor‐1α, MDA, and free ferrous ions in the villus tissue of the RSA group increased, while GPX4 levels were downregulated, suggesting that ferroptosis may be involved in the development of RSA [27]. Another investigation revealed lower GPX4 expression in placental trophoblasts from women with spontaneous abortion compared to those who underwent elective termination. At the same time, by establishing an in vitro natural abortion trophoblast cell model, it was found that the long non‐coding RNA *H19* contributes to natural abortion by promoting ferroptosis [28]. Our findings demonstrate pronounced ferroptosis in trophoblasts from both clinical RSA cases and experimental murine models, indicating its therapeutic targeting potential for this condition.

Key immune populations, including decidual macrophages and dendritic cells uphold immunological balance at the maternal‐fetal interface in early gestation. They not only participate in the proliferation and differentiation of endometrial stromal cells but also promote trophoblast invasion and vascular remodeling at the maternal‐fetal interface, serving as regulatory factors for embryo implantation, development, and placental function establishment [9]. Research indicates that ferroptosis‐mediated cell death releases deoxyadenosine monophosphate and lipid peroxides, which can influence macrophage polarization and lead to immune dysfunction [29, 30]. Decidual macrophages regulate trophoblast migration and invasion, as evidenced by current research [31]. Aberrant decidual macrophage‐trophoblast crosstalk causes trophoblastic dysfunction, which is closely associated with RSA [12]. However, whether decidual macrophages influence trophoblast ferroptosis in RSA remains unexplored. Next, integrating transcriptomic sequencing data, we found that decidual macrophages derived from RSA patients significantly promoted ferroptosis in trophoblasts. This led to impaired proliferation, migration, and invasion capabilities while accelerating trophoblast apoptosis. Furthermore, we observed that inhibiting ferroptosis reversed the suppression of trophoblast function by RSA‐derived decidual macrophages. RNA‐seq results identified *HMOX1* as the most significantly differentially expressed gene in the RSA‐derived decidual macrophage‐mediated ferroptosis signaling pathway. As the rate‐limiting enzyme in heme degradation, HMOX1 generates carbon monoxide (CO), biliverdin (BV), and ferrous ions. While CO and BV exert cytoprotective effects by scavenging ROS, the concomitant release of ferrous ions can paradoxically exacerbate ROS production via the Fenton reaction [32]. HMOX1 upregulation has been consistently linked to ferroptosis pathogenesis in prior research [33, 34, 35]. Consistent with these reports, we found that knocking down *HMOX1* in trophoblasts effectively reversed both the ferroptosis induced by RSA‐derived decidual macrophages and the resulting suppression of trophoblast function.

Macrophages modulate trophoblast function at the maternal‐fetal interface via secretion of immune mediators [36]. In this study, to investigate the key mediators by which macrophages at the maternal‐fetal interface regulate trophoblast ferroptosis in the involvement of RSA, analysis combined with transcriptome sequencing revealed that *CXCL2* was the gene with the most significant differential expression within the cytokine‐cytokine receptor interaction signaling pathway. Research indicates that CXCL2 is a key chemokine secreted by macrophages, released during tissue infection or injury as a mediator of the macrophage response to inflammatory stimuli [37]. According to some studies, *CXCL2*, as a ferroptosis‐related gene, can increase the susceptibility of liver cancer cells to ferroptosis [38]. CXCL2 exerts its pathophysiological effects through the CXCR2 [15]. Interestingly, our study found that decidual macrophages can regulate trophoblast ferroptosis and trophoblast function by secreting CXCL2 to upregulate CXCR2 expression on the trophoblast surface. Research indicates that CXCL2/CXCR2 signaling can activate multiple G protein‐mediated signaling cascades to enhance NF‐κB transcriptional activity [16, 17]. It has been reported that the promoter region of the *HMOX1* gene contains NF‐κB binding sites [18]. Additionally, the NF‐κB pathway was significantly enriched in trophoblast cells co‐cultured with RSA decidual macrophages. Further reversal experiments revealed that the CXCL2/NF‐κB/HMOX1 signaling axis serves as the key mechanism by which decidual macrophages regulate ferroptosis and function in RSA. Interactions occur between fetal trophoblasts and immune cells at the maternal‐fetal interface [39]. Our further research found that co‐cultured trophoblast cells with RSA‐derived decidual macrophages can promote macrophage pro‐inflammatory polarization by reducing IL‐6 secretion. Studies indicate that IL‐6 is a multifunctional cytokine involved in immune regulation, capable of activating JAK2/STAT3 pathway. Similarly, our study found that after co‐culturing with decidual macrophages derived from RSA patients, trophoblasts regulate macrophage inflammatory polarization by inhibiting the JAK2/STAT3 axis through IL‐6.

In addition, we found that compared with NP mice, the expression of HMOX1, CXCL2, and p‐p65 at the placental interface of AP mice was significantly increased. Therefore, we used AAV9 shRNA to knock down *HMOX1* or *CXCL2* genes *in vivo* or intervened with NF‐κB inhibitor PDTC ammonium in AP mice. The results showed that knocking down *HMOX1* and *CXCL2* genes or inhibiting NF‐κB in the fetal reduced embryo absorption rate, inhibited placental interface ferroptosis, and promoted the migration and invasion of trophoblast cells. These findings indicate the potential of targeted strategies targeting the CXCL2/NF‐κB/HMOX1 axis to inhibit ferroptosis in placental trophoblast cells in the clinical treatment of RSA. However, although we intervened with adeno‐associated viruses or inhibitors in mice, their effects were not limited to trophoblast cells, but also affected other cells, including macrophages and mesenchymal cells. The phenotype we observed may not only be caused by specific gene silencing in the uterus/placenta, but also by off‐target knockdown in the liver, ovaries, brain, or other organs that regulate the hypothalamic pituitary gonadal axis and pregnancy outcomes. This is a limitation of our study, and we hope to use uterine targeting strategies (such as intrauterine delivery, tissue‐specific promoters, or conditional genetic models) in future research to clarify whether the phenotype is caused by local uterine/placental regulation to address this issue.

According to reports, Eriodictyol, a natural flavonoid found in citrus fruits, possesses multiple biological activities, including antioxidant, anti‐inflammatory, and anti‐osteoclastogenic effects [40, 41, 42]. Research indicates that Eriodictyol can mitigate the pregnancy outcomes in mice that are aborted [23]. This aligns with the findings of our study, though it remains unclear whether it alleviates ferroptosis at the placental interface in mice with aborted pregnancies. Our evidence indicates that Eriodictyol effectively reduces ferroptosis at the placental interface in mice. While Eriodictyol treatment can decrease CXCL2 expression at the placental interface, further evidence is needed to validate how Eriodictyol exerts its therapeutic effects via CXCL2, and in this study, we used computer simulation docking technology to claim that Eriodictyol targets CXCL2, which is not rigorous enough. Our subsequent research can further obtain binding kinetic parameters through surface plasmon resonance and other methods to deepen our understanding of the details of the interaction between the two.

## CONCLUSION

In summary, we found that decidual macrophages in RSA patients can induce ferroptosis in trophoblast cells, suggesting that targeting this mechanism may offer new opportunities for reshaping maternal‐fetal tolerance. This study provides novel insights into the dysregulation of the maternal‐fetal interface immune microenvironment in RSA. Furthermore, it offers crucial theoretical support for developing RSA clinical intervention strategies focused on modulating decidual macrophage‐mediated trophoblast ferroptosis.

## METHODS

### Clinical sample collection

This study enrolled 15 patients with recurrent spontaneous abortion (RSA) and 15 patients with normal pregnancies (healthy control, HC), obtaining placental tissue from each group. All participants provided informed consent, and the study was approved by the Ethics Committee of Wuhan University Renmin Hospital (WDRY2021‐KS050). The inclusion and exclusion criteria and baseline data of the two groups of patients were as described previously [19]: the criteria for participation of normal pregnant women included: no history of adverse pregnancy outcomes, such as spontaneous abortion, fetal death or stillbirth; the inclusion criteria for RSA patients were: at least 2 consecutive unexplained miscarriages, and exclusion of known causes of miscarriage such as chromosomal abnormalities in both partners, female reproductive anatomical abnormalities, infectious factors, endocrine and metabolic disorders, thrombophilia (coagulation disorders, abnormal autoantibodies), embryonic chromosomal abnormalities and other known causes of miscarriage. The baseline characteristics of the patients are summarized in Table S1. Gently clean the obtained tissue with physiological saline, and after washing the tissue, completely separate the thin and soft pink villous tissue, leaving the rest as decidua tissue. The decidual tissue was washed with pre‐chilled sterile PBS and processed into a cell suspension. Cells were separated by density gradient centrifugation, and CD14‐positive magnetic bead sorting kit was used for the isolation of decidual macrophages.

### Analysis of GSH content and GPX activity

The tissue and cell samples were lysed using a 5% solution of 5‐sulfosalicylic acid. Cellular glutathione content was measured with a commercial Glutathione Assay Kit (354102, Sigma‐Aldrich) in accordance with the manufacturer's guidelines. Glutathione peroxidase (GPX) activity was assessed using the Glutathione Peroxidase Assay Kit (ab102530; Abcam), following the provided instructions.

### Iron determination

Measure Fe^2+^ content using the Iron Detection Kit (BC5415, Solarbio Life Science). All procedures were performed according to the manufacturer's protocol. Measure absorbance values at 593 nm using an enzyme‐linked immunosorbent assay reader.

### Malondialdehyde (MDA) measurement

MDA concentration detection employs the MDA assay kit (BC0025, Solarbio Life Science), adhering to the kit's operating procedures.

### Quantitative Real‐Time PCR

Total RNA was extracted using TRIzol (15596026, Thermo Fisher Scientific Co., Ltd.) and reverse transcribed with a reverse transcription kit (RR036A, Takara, Shiga, Japan). For mRNA expression analysis, the TB Green Premix ExTaq II (TliRNaseH Plus) kit (RR420A, Takara) was employed. All primer sequences are provided in Table S2.

### Western blotting

Proteins were extracted in RIPA buffer and separated by SDS‐PAGE. After transfer to PVDF, the membrane was blocked and incubated with primary antibody, followed by HRP‐conjugated secondary antibody. Bands were visualized using ECL and quantified in ImageJ. Details regarding the antibodies used are provided in Table S3.

### Immunohistochemistry

Fix the sample in 10% formaldehyde solution. As previously described, proceed with embedding, sectioning, dewaxing, rehydration, antigen retrieval, serum blocking, and incubation with the primary antibody overnight at 4°C, followed by incubation with horseradish peroxidase [10, 12].

### Cell culture and intervention

Human trophoblast cell line HTR‐8/SVneo (HTR‐8) cells were cultured in DMEM/F12 medium (Gibco). Primary decidual macrophages and THP‐1 cells were cultured in RPMI 1640 medium (Gibco). The HTR‐8 cell line and THP‐1 cell line were purchased from the servicebio (Wuhan). All cells were maintained in a humidified incubator at 37°C with 5% CO_2_.

HMOX1 shRNA, CXCL2 shRNA, HMOX1 overexpression plasmid, CXCL2 overexpression plasmid, p65 siRNA and negative control were purchased from Vigene (Jinan, Shandong, China). Transfection was performed using Lipofectamine 2000 Transfection Reagent (11668019, Thermo Fisher Scientific Inc.) according to the manufacturer's instructions.

The THP‐1 cell line was differentiated into M0 macrophages by treatment with 100 ng/ml phorbol 12‐myristate 13‐acetate (PMA, Sigma) for 24 h.

For the inhibition of ferroptosis, Ferrostatin‐1(60 nM, HY‐100579, MCE, Shanghai, China, dissolved in dimethyl sulfoxide) was used to treat cells for 48 h.

Inhibition of the NF‐κB pathway was achieved by treating cells with an NF‐κB inhibitor [pyrrolidinedithiocarbamate ammonium (PDTC ammonium) (10 µM, HY‐18738, MCE, dissolved in dimethyl sulfoxide)] for 48 h.

For activation of JAK2/STAT3 signaling, a STAT3 agonist ML115 (10 µM, HY‐111152, MCE, dissolved in dimethyl sulfoxide) was used to treat cells for 48 h.

For IL‐6 supplementation, the recombinant human IL‐6 (50 ng/mL, 200‐06, Peprotech, USA, dissolved in PBS) was added to treat cells for 48 h.

For CXCL2 supplementation, the recombinant human CXCL2 (10 ng/mL, 300‐39, Peprotech, dissolved in PBS) was added to treat cells for 24 h,48 h, and 72 h.

Eriodictyol (15 µM, HY‐N0637, MCE, dissolved in dimethyl sulfoxide) was used to treat cells for 48 h.

For activation of the NF‐κB pathway, NF‐κΒ activator 1 (5 µM, HY‐134476, MCE, dissolved in dimethyl sulfoxide) was used to treat cells for 48 h.

### Transcriptome sequencing

Total RNA was extracted from HTR‐8 cells in two groups (HC‐co and RSA‐co) with three biological replicates per group. The mRNA was purified and fragmented, cDNA was synthesized, PCR was used to enrich library fragments, library quality control was performed. Paired‐end sequencing was performed on an Illumina NovaSeq. 6000 platform (PersonalBio). Raw reads were filtered using Cutadapt (removal of adapters, at least 10 bp overlap, 20% mismatch allowed) and reads with average quality < Q20 were discarded. Clean reads per sample ranged from 45.2 to 51.3 million, with a mapping rate of 98.8% to the human reference genome (GRCh38) using HISAT2. Gene‐level counts were obtained with HTSeq. Fragments per kilobase of transcript per million mapped reads (FPKM) was used to normalize gene expression levels. Differential expression analysis was performed using DESeq2 with |log_2_FoldChange| > 1 and *p*‐value < 0.05. GO enrichment was conducted with topGO and KEGG enrichment with clusterProfiler (*p*‐value < 0.05). All analyses were performed on the Personalbio GenesCloud platform.

### ROS measurement

According to the manufacturer's instructions, intracellular ROS levels and mitochondrial superoxide production were assessed using the DCFH‐DA oxidative stress indicator (CA1420, Solarbio) and MitoSOX Red (MCE, HY‐D1055). Specifically, cells were cultured in serum‐free medium supplemented with the corresponding probes at 37°C for 30 min. Subsequently, cells were harvested, and fluorescence signals were measured by flow cytometry or photographed under a fluorescence microscope.

### Assessment of lipid peroxidation using C11‐BODIPY and Liperfluo staining

C11‐BODIPY staining: Incubate the processed cells with 5 μM C11‐BODIPY (D3861, Thermo Fisher Scientific) at 37°C for 20 min. Then collect the cells and analyze them by flow cytometry.

Liperfluo staining: Liperfluo Assay Kit (L248, DOJINDO, Japan) is used to measure lipid peroxide levels. Experiments are conducted according to a specific protocol, with observations made using a fluorescence microscope.

### Mitochondrial membrane potential assay

Mitochondrial membrane potential was analyzed using the JC‐1 fluorescent probe (M8650, Solarbio). Briefly, cells were incubated with JC‐1 solution (1 mL) at 37°C for 20 min (dark conditions), followed by two buffer washes. Fluorescence signals were captured after replacing with fresh culture medium.

### CCK‐8

Cell viability was assessed using the Cell Counting Kit‐8 (CCK‐8) (C0038, Beyotime Biotechnology). Briefly, cells were seeded into a 96‐well plate, medium containing CCK‐8 reagent was added, and the mixture was incubated at 37°C for 2 h. The absorbance value at 450 nm was measured using a microplate reader.

### EdU assay

Cell proliferation was measured using the EdU Assay Kit (C0075S, Beyotime). In brief, cells were incubated with 1X EdU solution for 2 h, then fixed in 4% paraformaldehyde. Samples were stained with the reaction mixture from the EdU kit, and cell nuclei were stained with Hoechst 33,342 solution. Fluorescent images were captured under a fluorescence microscope.

### TUNEL

TUNEL assay (C1088, Beyotime) was performed to assess apoptosis. After fixation, cells were permeabilized (0.3% Triton X‐100, 5 min), incubated with TUNEL reaction mix (50 μL, 37°C, 60 min, dark). Finally, the samples were observed.

### Flow cytometry detection of apoptosis

Add 7‐AAD reagent and PE Annexin V reagent to the cell suspension in sequence (40310ES60, Yeasen). Gently mix, then incubate the cells at room temperature for 15 min for staining. Subsequently, analyze the apoptosis rate using flow cytometry.

### Wound healing assay

Allow cells in a six‐well plate to confluently grow. Using a sterile pipette tip, create a straight scratch perpendicular to the bottom of the plate. Gently wash the wells with PBS. After 48 h, observe wound closure and photograph under an inverted microscope to assess cell migration capacity.

### Cell invasion assay

Inoculate 200 μL of serum‐free cell suspension into the upper chamber, while adding 500 μL of fresh complete medium to the lower chamber of the Transwell insert. After incubation, fix with paraformaldehyde and stain with crystal violet.

### Enzyme‐linked immunosorbent assay (ELISA)

Cytokine levels (CXCL2(orb1289481, Biorbyt, United Kingdom) and IL‐6 (CSB‐E04638h, Cusabio)) were determined using ELISA kits according to the manufacturer's protocol. Samples and standards were incubated with the provided reagents, and absorbance was determined using a microplate reader.

### Immunofluorescence

Samples were fixed with 4% PFA (15 min), permeabilized with 0.3% Triton X‐100 (8 min) and blocked with 5% BSA (30 min). Primary antibodies were incubated overnight at 4°C. After washing, samples were incubated with secondary antibodies (30 min, dark conditions), then stained with DAPI, and images were recorded. Detailed antibody information can be found in Table S3.

### Flow cytometry analysis of macrophage polarization

Single cell suspensions were blocked for 10 min and then stained with fluorescent antibodies. Samples were analyzed using flow cytometry. Antibody details are provided in Table S3.

### Chromatin immunoprecipitation assay‐qPCR (CHIP‐qPCR)

Cells were crosslinked by 1% formaldehyde, lysed, and the chromatin was broken by ultrasound. The lysates were collected and incubated with agarose beads, which were pre‐conjugated with IgG or anti‐p65. After overnight incubation at 4°C, the beads were isolated and the DNA fragments were enriched. The abundance of HMOX1 promoter was then detected by qPCR methods.

### Dual‐luciferase reporter gene experiment

To validate the transcriptional activation of HMOX1 by NF‐κB, point mutations were introduced into the HMOX1 promoter, and luciferase reporter vectors were constructed for both the wild‐type(WT) and a mutant(MUT) version with a mutation in the p65‐binding sequence. The constructed vectors were transfected into cells, and the dual‐luciferase activity was detected by Dual‐Luciferase Reporter Assay System.

### Animal experiments

In vivo experiments were conducted using CBA/J females, DBA/2 males and BALB/c male mice. Four weeks prior to mating, female mice were exposed to intravenous injections of adeno‐associated virus (AAV)9, which carries small hairpin RNA against HMOX1 (AAV‐sh‐HMOX1), small hairpin RNA against CXCL2 (AAV‐sh‐CXCL2), coding sequence of HMOX1 (AAV‐OE‐HMOX1), coding sequence of CXCL2 (AAV‐OE‐CXCL2)or their corresponding negative control (AAV‐sh‐Ctrl or AAV‐OE‐Ctrl) (Genechem, Shanghai). CBA/J female mice were mated with BALB/c males (normal pregnancy, NP) or DBA/2 males (abortion‐prone pregnancy, AP). On Day 7.5 of pregnancy, Eriodictyol (40 mg/kg, HY‐N0637, MCE, dissolved in corn oil) was injected intraperitoneally. On day 8.5 and 10.5 of pregnancy, PDTC ammonium (40 mg/kg, HY‐18738, MCE, dissolved in corn oil)] or NF‐κΒ activator 1 (10 mg/kg, HY‐134476, MCE, dissolved in corn oil) was injected intraperitoneally. On Day 11.5 of pregnancy, mice were euthanized to determine embryo resorption rates and collect placental tissues. Animal experiments were approved by the Experimental Animal Welfare and Ethics Committee of the Renmin Hospital of Wuhan University (WDRM20220603B).

### Drug screening and docking

This study utilized the TCM‐ID database to screen out 10 herbal components targeting CXCL2. CB‐Dock2 was employed to simulate the binding of these compounds with CXCL2 and calculate their binding affinities.

### Statistical analysis

All data were presented as mean ± standard deviation (SD) from at least three independent experiments. Student's *t*‐tests were used to compare normal continuous variables between two groups. One‐way analysis of variance was used to compare differences for normal continuous variables among multiple groups, with a further Tukey post‐hoc test performed if the difference between groups was statistically significant. Simple linear regression was used to quantify the linear correlation between two continuous variables. *p < 0.05, **p < 0.01 were considered statistically significant.

## AUTHOR CONTRIBUTIONS

**Xin Chen**: Writing—original draft; data curation; software; conceptualization; investigation; visualization; funding acquisition. **Xueqin Ma**: Software. **Pengcheng Pang**: Visualization. **Heng Zhou**: Data curation. **Ruohan Li**: Conceptualization. **Yan He**: Software; resources; investigation; visualization. **Yan Zhang**: Project administration; investigation; validation. **Jing Yang**: Writing—review and editing; writing—original draft; formal analysis. **Qianlin Song**: Project administration; methodology; supervision. **Qingsong Ye**: Supervision; funding acquisition; data curation; software; conceptualization; investigation; visualization; writing—review and editing.

## CONFLICT OF INTEREST STATEMENT

The authors declare no conflicts of interest.

## ETHICS STATEMENT

The study has obtained informed consent from the patients and has been approved by the Ethics Committee of the Renmin Hospital of Wuhan University (WDRY2021‐KS050). Animal experiments were approved by the Experimental Animal Welfare and Ethics Committee of the Renmin Hospital of Wuhan University (WDRM20220603B).

## Supporting information


**Figure S1:** Decidual macrophages derived from patients with RSA cause ferroptosis in trophoblasts.
**Figure S2:** Blocking ferroptosis successfully reversed the impairment of trophoblast function by decidual macrophages in RSA patients.
**Figure S3:** RSA‐derived decidual macrophages regulate trophoblast ferroptosis via HMOX1.
**Figure S4:** RSA‐derived decidual macrophages produce CXCL2 as a key regulator in the ferroptosis and other functions of trophoblast.
**Figure S5:** NF‐κB directly regulates *HMOX1* transcription.
**Figure S6:** NF‐κB pathway expression in villous tissues from HC and RSA patients.
**Figure S7:** The NF‐κB signaling pathway mediates trophoblast ferroptosis induced by RSA‐derived decidual macrophages.
**Figure S8:** HMOX1 is a functional downstream effector of NF‐κB that drives ferroptosis and functional impairment in trophoblast cells.
**Figure S9:** HMOX1 is an indispensable downstream effector of CXCL2.
**Figure S10:** RSA‐derived decidual macrophage‐educated trophoblasts promote macrophage inflammatory activation.
**Figure S11:** Trophoblast cells co‐cultured with decidual macrophages from RSA patients may influence macrophage polarization.
**Figure S12:** Using the TCM‐ID database, herbal components targeting aborted were screened and identified.
**Figure S13:** Eriodictyol alleviates ferroptosis at the maternal‐fetal interface in a mouse model of abortion.
**Figure S14:** Eriodictyol exerts its inhibitory effect on miscarriage through the CXCL2/NF‐κB/HMOX1 axis.

**Table S1:** Characteristics of women from the healthy control (HC) and recurrent spontaneous abortion (RSA) groups [19].
**Table S2:** The primer sequences used in the study.
**Table S3:** The specific antibodies used for Western blotting, immunohistochemistry, immunofluorescence staining, and flow cytometry.

## Data Availability

The data that support the findings of this study are available on request from the corresponding author. The data are not publicly available due to privacy or ethical restrictions. Transcriptomic data have been deposited in the NCBI BioProject database under accession number:PRJNA1457674 https://www.ncbi.nlm.nih.gov/bioproject/1457674). No original code was generated in this study. The data used are saved in GitHub (https://github.com/Ferroptosis2026287/Figure-data-2026.git). Supplementary materials (methods, figures, tables, graphical abstract, slides, videos, Chinese translated version, and updated materials) may be found in the online DOI or iMeta Science http://www.imeta.science/.
