# Supplementary material for: Decidual macrophage‐mediated ferroptosis in trophoblasts leads to recurrent spontaneous abortion
Source: Imeta. 2026 Jun 14;5(3):e70138. doi: 10.1002/imt2.70138 (PMC13377409; doi:10.1002/imt2.70138)
Supplement: Supplementary file 1 — Figure S1: Decidual macrophages derived from patients with RSA cause ferroptosis in trophoblasts. Figure S2: Blocking ferroptosis successfully reversed the impairment of trophoblast function by decidual macrophages in RSA patients. Figure S3: RSA‐derived decidual macrophages regulate trophoblast ferroptosis via HMOX1. Figure S4: RSA‐derived decidual macrophages produce CXCL2 as a key regulator in the ferroptosis and other functions of trophoblast. Figure S5: NF‐κB directly regulates HMOX1 transcription. Figure S6: NF‐κB pathway expression in villous tissues from HC and RSA patients. Figure S7: The NF‐κB signaling pathway mediates trophoblast ferroptosis induced by RSA‐derived decidual macrophages. Figure S8: HMOX1 is a functional downstream effector of NF‐κB that drives ferroptosis and functional impairment in trophoblast cells. Figure S9: HMOX1 is an indispensable downstream effector of CXCL2. Figure S10: RSA‐derived decidual macrophage‐educated trophoblasts promote macrophage inflammatory activation. Figure S11: Trophoblast cells co‐cultured with decidual macrophages from RSA patients may influence macrophage polarization. Figure S12: Using the TCM‐ID database, herbal components targeting aborted were screened and identified. Figure S13: Eriodictyol alleviates ferroptosis at the maternal‐fetal interface in a mouse model of abortion. Figure S14: Eriodictyol exerts its inhibitory effect on miscarriage through the CXCL2/NF‐κB/HMOX1 axis. [file IMT2-5-e70138-s002.docx]

**Supporting Information to**

**Decidual macrophage-mediated ferroptosis in trophoblasts leads to recurrent spontaneous abortion**

**Running title**: Decidual macrophage-induced ferroptosis in trophoblasts causes RSA

Xin Chen ^1, 2^, Xueqin Ma ^1, 2^, Pengcheng Pang^3^, Heng Zhou^3^, Ruohan Li^4^, Yan He^5^ ^*^, Yan Zhang ^1 *^, Jing Yang ^1, 6^ ^*^, Qianlin Song ^2, 7^ ^*^, Qingsong Ye^3, 8^ ^*^

^1^ Department of Obstetrics and Gynecology, Renmin Hospital of Wuhan University, Wuhan, Hubei 430060, China

^2^ Central Laboratory, Renmin Hospital of Wuhan University, Wuhan, Hubei 430060, China

^3^ Center of Regenerative Medicine, Department of Stomatology, Renmin Hospital of Wuhan University, Wuhan, Hubei 430060, China

^4^ Faculty of Medicine and Health, The Otago University, Dunedin, 9016, New Zealand

^5^ School of Stomatology, Guangxi Medical University, Nanning, Guangxi, 530021, China

^6^ Reproductive Medical Center, Renmin Hospital of Wuhan University and Hubei Clinic Research Center for Assisted Reproductive Technology and Embryonic Development, Wuhan, Hubei 430060, China

^7^ Department of Urology, Renmin Hospital of Wuhan University, Wuhan, Hubei 430060, China

^8^ School of Dentistry, The University of Sydney, NSW, 2006, Australia

**^*^ Corresponding author:** qingsongye@whu.edu.cn (Qingsong Ye); qlsong2018@whu.edu.cn (Qianlin Song); [dryangjing@whu.edu.cn](mailto:dryangjing@whu.edu.cn) (Jing Yang); rm002125@whu.edu.cn(Yan Zhang); helen-1101@hotmail.com (Yan He)

**Figure S1.** **Decidual macrophages derived from patients with RSA cause ferroptosis in trophoblasts. (A)** Mean fluorescence intensity (MFI) ratio (red/green) of JC-1. **(B**–**E)** The mRNA expression of ferroptosis-related markers *ACSL4*, *TFR1*, *SLC7A11*, and *GPX4* was detected by qRT-PCR. Student's *t*-test was employed for comparisons between two groups. ***p* < 0.01.

**Figure S2. Blocking ferroptosis successfully reversed the impairment of trophoblast function by decidual macrophages in RSA patients.** **(A)** Cell proliferation capacity was assessed using the CCK-8 assay. **(B)** Cell proliferation capacity was assessed using the EdU assay. **(C)** Detection of apoptosis via TUNEL staining. **(D)** Flow cytometric analysis and statistical results of cell apoptosis. **(E)** Assessment of trophoblast cell migration capacity through wound healing assays. **(F–G)** Evaluation of trophoblast cell invasion capacity via Transwell assays. **(H)** Detection of mRNA expression levels for apoptosis markers *BAX* and *Bcl2*, and EMT markers *E-cadherin* and *Vimentin* via qRT-PCR. **(I)** The expression levels of key proteins involved in apoptosis (BAX, Bcl-2) and EMT (E-cadherin, Vimentin) were evaluated using Western blot. Human primary decidual macrophages were seeded in the upper chamber, while HTR-8/SVneo (HTR-8) trophoblast cell line was seeded in the lower chamber and ferroptosis inhibitor Ferrostatin-1 (Fer-1, 60nM) were added. The cells were then co cultured in a cell culture incubator for 48 hours, **(J)** CCK-8 assay was employed to measure cell proliferation. **(K)** We employed an EdU assay to quantify cell proliferation. **(L)** The TUNEL assay was utilized to detect apoptotic cells. **(M)** Flow cytometric analysis of cell apoptosis. **(N)** Trophoblast cell migration capacity was determined via the wound-healing assay. **(O)** Transwell assay for trophoblast invasion capacity. **(P)** Analysis of apoptosis- and EMT-related gene expression (*BAX*, *Bcl-2*, *E-cadherin*, *Vimentin*) was conducted via qRT-PCR. **(Q)** Western blot for protein expression of apoptosis markers BAX and Bcl2, and EMT markers E-cadherin and Vimentin. Student's *t*-test and one-way ANOVA were employed for comparisons between two groups and multiple groups, respectively. ***p* < 0.01, ns: not significant.

**Figure S3.** **RSA-derived decidual macrophages regulate trophoblast ferroptosis via HMOX1. (A–B)** Protein expression of HMOX1 in both groups detected by Western blot. **(C)** Quantitative MFI values for DCFH-DA. **(D)** Quantitative MFI values for BODIPY 581/591 C11. **(E)** Quantitative MFI of mitoSOX in trophoblast cells. **(F)** Quantitative MFI values of Liperfluo staining. **(G–J)** Levels of GSH, GPx activity, Fe²⁺, and MDA in trophoblast cells. **(K)** The mRNA expression of ferroptosis-related markers *ACSL4*, *TFR1*, *SLC7A11*, and *GPX4* was detected by qRT-PCR. **(L)** Statistical values of mitochondrial membrane potential assessed by JC-1 staining. **(M)** Cell proliferation capacity assessed by CCK8 assay. **(N)** Apoptosis statistical values determined by TUNEL staining. **(O)** Migration capacity of trophoblast cells evaluated by wound healing assay. **(P)** The mRNA expression of apoptosis markers *BAX* and *Bcl2*, and EMT markers *E-cadherin* and *Vimentin*, detected by qRT-PCR. Student's *t*-test and one-way ANOVA were employed for comparisons between two groups and multiple groups, respectively. **p* < 0.05, ***p* < 0.01, ns: not significant.

**Figure S4.** **RSA-derived decidual macrophages produce CXCL2 as a key regulator in the ferroptosis and other functions of trophoblast.** **(A)** The mRNA expression of *CXCR2* was detected by qRT-PCR. **(B–C)** Protein expression of CXCR2 in the two groups detected by Western blot. **(D–E)** Protein expression of CXCL2 in the two groups detected by Western blot. **(F)** MFI quantification values from Liperfluo staining. **(G–J)** Levels of GSH, GPx activity, Fe²⁺, and MDA in trophoblast cells. **(K)** The mRNA expression of ferroptosis-related markers *ACSL4*, *TFR1*, *SLC7A11*, and *GPX4* was detected by qRT-PCR. **(L)** Statistical values of the protein expression of ferroptosis-related proteins ACSL4, TFR1, SLC7A11, and GPX4. **(M)** Cell proliferation capacity was detected by the CCK8 assay. **(N)** Statistical analysis of apoptosis detected by TUNEL staining. **(O)** Statistical analysis of apoptosis by flow cytometry. **(P)** Statistical analysis of trophoblast cell migration capacity by wound healing assay. **(Q)** Detection of mRNA expression of apoptosis markers *BAX* and *Bcl2*, and EMT markers *E-cadherin* and *Vimentin* by qRT-PCR. Student's *t*-test and one-way ANOVA were employed for comparisons between two groups and multiple groups, respectively. ***p* < 0.01, ns: not significant.

**Figure S5.** **NF-κB directly regulates *HMOX1* transcription. (A–B)** CHIP-qPCR showed the binding relationship between NF-κB and *HMOX1* promoter. For CXCL2 supplementation, the recombinant human CXCL2 (10ng/mL) was added to treat cells for 48 hours. **(C)** The regulation of *HMOX1* transcription by NF-κB was analyzed using a dual luciferase reporter assay. One-way ANOVA was employed for comparisons between multiple groups. ***p* < 0.01, ns: not significant.

**Figure S6.** **NF-κB pathway expression in villous tissues from HC and RSA patients. (A–B)** IHC detection of p-p65 protein expression in the two groups. Student's *t*-test was employed for comparisons between two groups. ***p* < 0.01.

**Figure S7.** **The NF-κB signaling pathway mediates trophoblast ferroptosis induced by RSA-derived decidual macrophages. (A–D)** Detection of the mRNA expression of ferroptosis-related markers *ACSL4*, *TFR1*, *SLC7A11*, and *GPX4* via qRT-PCR. Student's *t*-test was employed for comparisons between two groups. ***p* < 0.01.

**Figure S8. HMOX1 is a functional downstream effector of NF-κB that drives** **ferroptosis and functional impairment in trophoblast cells. (A)** Protein expression of p65 in both groups detected by Western blot. **(B)** The mRNA expression of *HMOX1* was detected by qRT-PCR. **(C–D)** Protein expression of HMOX1 in the two groups detected by Western blot. **(E)** Cellular ROS levels were measured via flow cytometry based on the mean fluorescence intensity (MFI) of DCFH-DA. **(F)** Intracellular lipid peroxidation was assessed using Lipofluo staining. **(G–J)** Levels of GSH, GPx activity, Fe²⁺, and MDA in trophoblast cells. **(K)** Western blot analysis and statistical values for ferroptosis makers ACSL4, TFR1, SLC7A11, and GPX4. **(L)** Cell proliferation capacity was detected by the CCK8 assay. **(M)** Apoptosis detected by TUNEL staining. **(N)** Determination of trophoblast cell migration capacity via wound healing assay. **(O)** Assessment of trophoblast cell invasion capacity via Transwell assay. **(P)** Western blot analysis was performed to evaluate the expression of apoptosis-related proteins (BAX and Bcl-2) and EMT markers (E-cadherin and Vimentin). Student's *t*-test was employed for comparisons between two groups. ***p* < 0.01.

**Figure S9. HMOX1 is an indispensable downstream effector of CXCL2. (A)** NF-κB pathway expression was compared in trophoblast cells by Western blot. **(B)** Protein expression of HMOX1 in the two groups detected by Western blot. **(C)** NF-κB pathway expression was compared in trophoblast cells by Western blot. For CXCL2 supplementation, the recombinant human CXCL2 (10ng/mL) was added to treat cells for 24 hours,48 hours, and 72 hours.**(D)** Protein expression of HMOX1 was detected by Western blot. For CXCL2 supplementation, the recombinant human CXCL2 (10ng/mL) was added to treat cells for 24 hours,48 hours, and 72 hours. **(E)** Protein expression of HMOX1 was detected by Western blot. For CXCL2 supplementation, the recombinant human CXCL2 (10ng/mL) was added to treat cells for 48 hours. **(F)** Cellular ROS levels were measured via flow cytometry based on the mean fluorescence intensity (MFI) of DCFH-DA. **(G)** Intracellular lipid peroxidation was assessed using Lipofluo staining. **(H–K)** Levels of GSH, GPx activity, Fe²⁺, and MDA in trophoblast cells. **(L)** Western blot analysis and statistical values for ferroptosis makers ACSL4, TFR1, SLC7A11, and GPX4. **(M)** Cell proliferation capacity was detected by the CCK8 assay. **(N)** Apoptosis detected by TUNEL staining. **(O)** Determination of trophoblast cell migration capacity via wound healing assay. **(P)** Assessment of trophoblast cell invasion capacity via Transwell assay. **(Q)** Western blot analysis was performed to evaluate the expression of apoptosis-related proteins (BAX and Bcl-2) and EMT markers (E-cadherin and Vimentin). Student's *t*-test and one-way ANOVA were employed for comparisons between two groups and multiple groups, respectively. ***p* < 0.01.

**Figure S10. RSA-derived decidual macrophage-educated trophoblasts promote macrophage inflammatory activation. (A)** MFI of CD86 and quantification values were examined by flow cytometry. **(B)** MFI of CD206 and quantification values were examined by flow cytometry. **(C)** MFI of CD86 and quantification values were examined by flow cytometry. For IL-6 supplementation, the recombinant human IL-6 (50ng/mL) was added to treat cells for 48 hours. **(D)** MFI of CD206 and quantification values were examined by flow cytometry. For IL-6 supplementation, the recombinant human IL-6 (50ng/mL) was added to treat cells for 48 hours. **(E)** MFI of CD86 and quantification values were examined by flow cytometry. For activation of JAK2/STAT3 signaling, a STAT3 agonist ML115 (10µM) was used to treat cells for 48 hours.**(F)** MFI of CD206 and quantification values were examined by flow cytometry. For activation of JAK2/STAT3 signaling, a STAT3 agonist ML115 (10µM) was used to treat cells for 48 hours. Student's *t*-test was employed for comparisons between two groups. ***p* < 0.01.

**Figure S11. Trophoblast cells co-cultured with decidual macrophages from RSA patients may influence macrophage polarization. (A)** Co-culture of trophoblast cells from HC and RSA patients with decidual macrophages, followed by co-culture with primary decidual macrophages, then detection of macrophages. **(B)** The mRNA expression levels of M1 phenotype markers (*TNF-α*, *CXCL9*, and *CD86*) and M2 phenotype markers (*CD206*, *CCL8*, and *CD163*) in each group were detected by qPCR. Student's *t*-test was employed for comparisons between two groups. ***p* < 0.01.

**Figure S12.** **Using the TCM-ID database, candidate herbal components predicted to interact with CXCL2 were screened and identified. (A)** Simulated binding image of Chlorogenic Acid with CXCL2 in CB-Dock2. **(B)** Simulated binding image of Cuminaldehyde with CXCL2 in CB-Dock2. **(C)** Simulated binding image of Ergosterol with CXCL2 in CB-Dock2. **(D)** Simulated binding image of Esculetin with CXCL2 in CB-Dock2. **(E)** Simulated binding image of Eucalyptol with CXCL2 in CB-Dock2. **(F)** Simulated binding image of Geraniol with CXCL2 in CB-Dock2. **(G)** Hyperoside binding to CXCL2 simulated in CB-Dock2. **(H)** Isoalantolactone binding to CXCL2 simulated in CB-Dock2. **(I)** Nerolidol binding to CXCL2 simulated in CB-Dock2.

**Figure S13. Eriodictyol alleviates ferroptosis at the maternal-fetal interface in a mouse model of abortion. (A)** Table of simulated binding affinity scores for drugs and CXCL2 in CB-Dock2. **(B)** Simulated binding images and magnification of Eriodictyol and CXCL2 in CB-Dock2. **(C–D)** Embryo resorption rates in two groups of mice after Eriodictyol intervention [On day 7.5 of pregnancy, Eriodictyol (40 mg/kg) was injected intraperitoneally]. **(E–F)** Validation of CXCL2 expression in placental tissues from both groups of pregnant mice via Western blot. **(G–H)** Validation of CXCL2 expression in placental tissues from both groups of pregnant mice via IHC. **(I–M)** Western blot quantification and statistical analysis of ferroptosis regulators ACSL4, TFR1, SLC7A11, and GPX4. **(N–Q)** Levels of GSH, GPx activity, Fe²⁺, and MDA at the placental interface. **(R–V)** Western blot quantification and statistical analysis of apoptosis (BAX, Bcl2) and EMT (E-cadherin, Vimentin) markers. Student's *t*-test was employed for comparisons between two groups. ***p* < 0.01.

**Figure S14. Eriodictyol exerts its inhibitory effect on miscarriage through the CXCL2/NF-κB/HMOX1 axis. (A)** Representative Co-immunoprecipitation images. **(B)** Protein expression of the NF-κB pathway and HMOX1 was compared in trophoblast cells by Western blot. **(C)** Protein expression of CXCL2 was detected in trophoblast cells by Western blot. **(D, F, and H)** Expression and statistical analysis of ferroptosis-related proteins: ACSL4, TFR1, SLC7A11, and GPX4, as determined by western blot. **(E, G, and I)** Protein expression and statistical analysis of apoptosis markers BAX and Bcl2, and EMT markers E-cadherin and Vimentin via Western blot. **(J–L)** Embryo resorption rates in both groups. **(A–B and D–I)** Eriodictyol (15 µM) was used to treat cells for 48 hours. **(F–G)** For activation of NF-κB pathway, NF-κΒ activator 1 (5 µM) was used to treat cells for 48 hours. **(J–L)** On day 7.5 of pregnancy, Eriodictyol (40 mg/kg) was injected intraperitoneally. **(K)**On day 8.5 and 10.5 of pregnancy, NF-κΒ activator 1 (10 mg/kg) was injected intraperitoneally. Student's *t*-test was employed for comparisons between two groups. ***p* < 0.01.
